# Distributed quantum sensing with measurement-after-interaction strategies

**DOI:** 10.1038/s41534-026-01224-z

**Published:** 2026-04-03

**Authors:** Jiajie Guo, Shuheng Liu, Matteo Fadel, Qiongyi He

**Affiliations:** 1 State Key Laboratory of Artificial Microstructure and Mesoscopic Physics, School of Physics, Frontiers Science Center for Nano-optoelectronics, Collaborative Innovation Center of Quantum Matter, Peking University, Beijing, China; 2 Department of Physics, ETH Zürich, Zürich, Switzerland; 3 Collaborative Innovation Center of Extreme Optics, Shanxi University, Taiyuan, Shanxi China; 4 Peking University Yangtze Delta Institute of Optoelectronics, Nantong, Jiangsu China; 5 Hefei National Laboratory, Hefei, China

**Keywords:** Optics and photonics, Physics

## Abstract

We investigate multiparameter quantum estimation protocols based on measurement-after-interaction (MAI) strategies, in which the probe state undergoes an additional evolution prior to linear measurements. As we show in our study, this extra evolution enables different level of advantages depending on whether it is implemented locally or nonlocally across the sensing nodes. By benchmarking MAI strategies in both discrete- and continuous-variable systems, we show that they can significantly enhance multiparameter sensitivity and robustness against detection noise, particularly when non-Gaussian probe states are employed, cases where standard linear measurements are often insufficient. We also derive analytical results for multiparameter squeezing and establish the corresponding scaling laws for spin-squeezed states, demonstrating that MAI protocols can reach the Heisenberg scaling. These results can be implemented in state-of-the-art experimental platforms involving atomic ensembles or optical fields.

## Introduction

Distributed quantum sensing aims at simultaneously estimate multiple parameters locally encoded in different nodes^1–3^. Recently, it has been attracting increasing interest due to its relevance to quantum networks^2–5^ and promising applications in a variety of technological applications, such as international clock synchronization^6^ and local beam tracking^7^. These perspectives lead to intensive investigations on how to enhance the multiparameter sensitivity beyond the classical limit: Various theoretical efforts focus on optimizing the use of entanglement and measurements to achieve the maximum allowed multiparameter sensitivity^3,5,8,9^. In addition, quantum-enhanced multiparameter sensitivity have been demonstrated in both continuous- and discrete-variable experiments^10–16^.

**Fig. 1:**
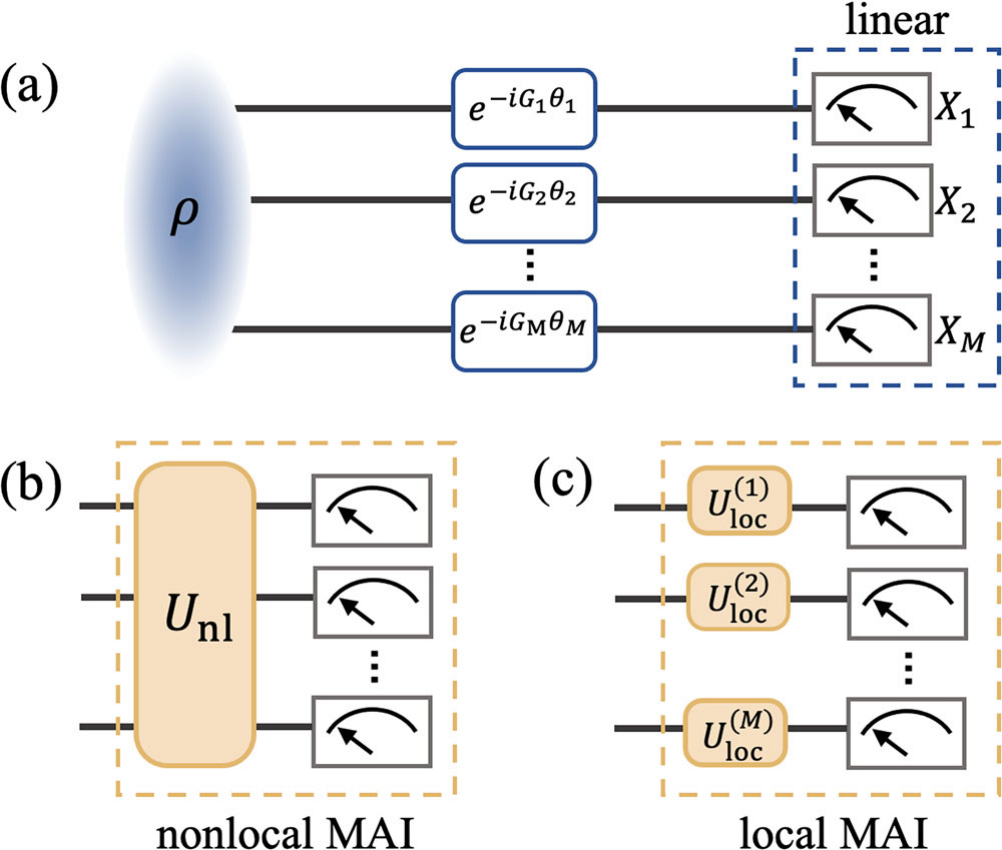
Illustration of distributed quantum sensing under different measurement strategies. A set of multiple phases ${\boldsymbol{\theta }}=({\theta }_{1},\cdots \,,{\theta }_{M})$ are locally encoded to the probe state *ρ*, where *G*_*m*_ are commuting phase generators. Then the multiparameter sensitivity will be estimated based on the results from (**a**) a set of local linear measurements *X*_*m*_. In the MAI protocol, (**b**) nonlocal evolutions *U*_nl_ or (**c**) local evolutions ${U}_{\rm{loc}}^{(m)}$ are introduced before the linear measurements.

In typical multiparameter estimation protocols, linear measurements, including collective spin observables in atomic ensembles and phase-space quadratures in optical fields, are performed on the probe states in order to estimate the unknown parameters^4,11,14,16,17^, see Fig. 1a. However, this measurement strategy, although experimentally easy to implement, comes at the cost of reduced detection capability. First- and second-order moments of the probability distribution of linear measurements readouts are, in fact, insensitive to non-Gaussian properties of the probe states^18^, which could provide a significant additional advantage in the metrological task. For this reason, taking inspiration from single parameter estimation scenarios, we introduce the measurement-after-interaction (MAI) strategy to distributed sensing tasks. This approach consists of adding an additional state evolution before the linear measurements to achieve an increased signal-to-noise ratio^19–25^. Without additional measurement statistics, the MAI strategy leads to enhanced noise robustness^26,27^ as well as in revealing the high sensitivity of non-Gaussian probe states^28,29^, as demonstrated in remarkable experiments.

When it comes to the framework of multiparameter estimation, we propose and compare two complementary scenarios: nonlocal and local MAI strategies. In the case of nonlocal MAI, see Fig. 1b, the additional state evolution is implemented on the whole probe state. However, since in many distributed sensing tasks the individual nodes can be far apart, a more practical strategy could be a local MAI protocol, see Fig. 1c, where the additional evolution is individually implemented on each node. The question is on how these protocols compare in terms of the achievable multiparameter estimation sensitivity and noise robustness.

In this work, we provide a general framework to estimate multiparameter sensitivity using MAI strategies. We benchmark both nonlocal and local protocols in both discrete- and continuous-variable systems, and demonstrate that both protocols show enhanced detection capability compared to the typical scenario solely relying on linear measurements. To consider experimentally relevant situations, we first analyze the paradigmatic case of multi-mode spin-squeezed states generated from the one-axis-twisting interaction, where the multiparameter sensitivity is determined by both mode entanglement and particle entanglement^8^. We show that the nonlocal MAI strategy is more powerful to capture mode entanglement compared to its local counterpart. Furthermore, we analytically derive the scaling laws under different measurement strategies, and surprisingly find that besides nonlocal MAI protocol, the local MAI protocol with small mode numbers is able to achieve the Heisenberg scaling. In addition, we further investigate two-mode squeezed vacuum states, as a simple case of continuous-variable Gaussian states, and show that the robustness against detection noises is significantly improved in MAI strategies.

## Results

### Distributed quantum sensing and the multiparameter squeezing matrix

The distributed sensing scenario can be described by a multimode interferometer, see Fig. 1, where in each of the *M* paths it is encoded an unknown phase *θ*_*m*_. The goal is then to estimate an arbitrary linear combination of these phases, *Θ* = **n**^*T*^***θ***, where **n** = (*n*_1_, ⋯ , *n*_*M*_), ∣**n**∣ = 1, specifies the linear combination and ${\boldsymbol{\theta}}=({\theta}_{1},\cdots \,,{\theta}_{M})$. These parameters are encoded on the probe state via the unitary evolution *ρ*(***θ***) = *e*^−*i***G*θ***^*ρe*^*i***G*θ***^, where ${\bf{G}}=({G}_{1},\cdots \,,{G}_{M})$ is a vector of local generators that commute with each other. After a sequence of *ν* independent measurements performed on the probe *ρ*(***θ***), an unbiased estimator ***θ***_est_ = (*θ*_est,1_, ⋯ , *θ*_est,*M*_) for ***θ*** can be constructed from the readouts. To represent the error of the estimation, a covariance matrix **Σ** with elements **Σ**_*ij*_ = Cov(*θ*_est,*i*_, *θ*_est,*j*_) is defined, which yields the variance of the linear combination of multiparameter estimation Var[*Θ*_est_] = **n**^*T*^**Σn** with *Θ*_est_ = **n**^*T*^***θ***_est_. Due to the fundamental limits given by the Cram$\acute{e}$r-Rao and quantum Cram$\acute{e}$r-Rao bounds^30–32^, it holds the hierarchy of sensitivities ${\boldsymbol{\Sigma }}\ge {{\bf{F}}}^{-1}/\nu \ge {{\bf{F}}}_{Q}^{-1}/\nu$, where **F** and **F**_*Q*_ are classical and quantum Fisher information matrix, respectively.

One of the commonly used estimator is known as the method of moments, which consists of estimating the unknown parameter from a moment of the measurement probability distribution. Let us consider a family of accessible local measurement operators ${\bf{X}}=({X}_{1},\cdots \,,{X}_{M})$, from which we express the moment-based covariance matrix as

1 $${\boldsymbol{\Sigma }}={(\nu {\bf{M}}[\rho ,{\bf{G}},{\bf{X}}])}^{-1},$$

with the moment matrix

2 $${\bf{M}}[\rho ,{\bf{G}},{\bf{X}}]={\bf{C}}{[\rho ,{\bf{G}},{\bf{X}}]}^{T}{\mathbf{\Gamma }}{[\rho ,{\bf{X}}]}^{-1}{\bf{C}}[\rho ,{\bf{G}},{\bf{X}}].$$

Here, **Γ**[*ρ*, **X**] is the covariance matrix with elements ${({\mathbf{\Gamma }}[\rho ,{\bf{X}}])}_{ij}={\rm{C}}{\rm{o}}{\rm{v}}({X}_{i},{X}_{j})$, and **C**[*ρ*, **G**, **X**] is the commutator matrix with elements ${({\bf{C}}[\rho ,{\bf{G}},{\bf{X}}])}_{ij}=-i\langle [{X}_{i},{G}_{j}]\rangle$.

The shot-noise limit is given as ${{\boldsymbol{\Sigma }}}_{\rm{SN}}={(\nu {{\bf{F}}}_{\rm{SN}}[{\bf{G}}])}^{-1}$, where ${{\bf{F}}}_{\rm{SN}}$ is the quantum Fisher information matrix determining the sensitivity limit from a classical scheme. To quantify the quantum-enhanced sensitivity above such classical limits, we make use of the squeezing matrix

3 $${\Xi }^{2}[\rho ,{\bf{G}},{\bf{X}}]={{\bf{F}}}_{\rm{SN}}{[{\bf{G}}]}^{\frac{1}{2}}{\bf{M}}{[\rho ,{\bf{G}},{\bf{X}}]}^{-1}{{\bf{F}}}_{\rm{SN}}{[{\bf{G}}]}^{\frac{1}{2}}.$$

Observing ${\Xi }^{2}[\rho ,{\bf{G}},{\bf{X}}] < {{\mathbb{I}}}_{M}$ reveals a quantum-enhanced sensitivity in the multiparameter scenario, i.e., ${\boldsymbol{\Sigma }} < {{\boldsymbol{\Sigma }}}_{{\rm{S}}{\rm{N}}}$.

### Nonlocal and local measurement-after-interaction (MAI) protocols

In typical experimental platforms for metrology, the moment-based sensitivity is determined by performing linear measurements, such as collective spin observables in atomic systems or quadrature operators in light fields. Although these linear measurements are optimal to reveal the sensitivity of Gaussian states undergoing Gaussian transformations, they are insufficient for non-Gaussian states. The high metrological potential of these states, in fact, resides in high-order moments that are more difficult to access.

An experimentally practical approach to effectively measure these high moments without the need to change detection apparatus consists of the measurement-after-interaction (MAI) protocol^19–22^. In this protocol, the probe state will go through a unitary evolution *U*(*τ*) = *e*^−*iHτ*^, with *H* a nontrivial Hamiltonian, before it is probed by linear measurements. The MAI protocol has been widely investigated in single-parameter estimation tasks, showing its advantages on improving the robustness against detection noise as well as revealing sensitivity in non-Gaussian states^24–29^. For multi-parameter estimation, numerical studies for an atomic ensemble where each atom carries an independent phase have suggested that MAI strategies can provide an advantage^33^. In the following, we provide a completely general framework for exploring the application of MAI methods in a distributed sensing scenario, providing analytical results.

First, let us introduce a family of accessible linear operators on the *m*-th mode, ${\mathcal{L}}=({L}_{1},\cdots \,,{L}_{K})$. In the typical scheme as in Fig. 1a, both phase generators and measurements are considered to be linear operators. Therefore, they can be expressed as ${G}_{m}={G}_{\rm{L}}^{(m)}:={{\bf{g}}}_{m}^{T}{\mathcal{L}}$ and ${X}_{m}={X}_{\rm{L}}^{(m)}:={{\bf{x}}}_{m}^{T}{\mathcal{L}}$, respectively, where **g**_*m*_, **x**_*m*_ are normalized real vectors. Then, based on the specific requirements of the considered estimation task, we investigate two possible measurement strategies: nonlocal MAI and local MAI, which are illustrated in Fig. 1b, c, respectively.

In a nonlocal MAI strategy, a unitary evolution ${U}_{\rm{nl}}={e}^{-i{H}_{\rm{nl}}\tau }$ is applied on the whole system before the local measurements are performed. This process is equivalent to performing a nonlocal MAI measurement on *m*-th mode, which reads

4 $${X}_{\rm{MAI},\rm{nl}}^{(m)}={U}_{\rm{nl}}^{\dagger }{X}_{\rm{L}}^{(m)}{U}_{\rm{nl}}.$$

In a local MAI strategy, on the other hand, we consider *M* unitary evolutions each performed on a single mode, i.e. ${U}_{\rm{loc}}^{(m)}={e}^{-i{H}_{\rm{loc}}^{(m)}{\tau }^{(m)}}$, such that the resulting local MAI operator can be expressed as

5 $${X}_{\rm{MAI},\rm{loc}}^{(m)}={U}_{\rm{loc}}^{(m)\dagger }{X}_{\rm{L}}^{(m)}{U}_{\rm{loc}}^{(m)}.$$

Note that, in general, these local unitary evolutions can be different from each other.

### Measurements optimization in MAI strategy

Given a vector of accessible linear operators on mode *m*, ${{\mathcal{L}}}^{(m)}=({L}_{1}^{(m)},\cdots \,,{L}_{K}^{(m)})$, we can further construct the global family by involving these local sets over all *M* modes, ${{\mathcal{A}}}_{\rm{L}}=({{\mathcal{L}}}^{(1)},\cdots \,,{{\mathcal{L}}}^{(M)})$. In the MAI strategy, an MAI vector ${{\bf{X}}}_{\rm{MAI}}^{(m)}$ is determined by an additional evolution *U*(*τ*), whose elements can be expressed as ${{\bf{X}}}_{\rm{MAI},k}^{(m)}={U}^{\dagger }(\tau ){L}_{k}^{(m)}U(\tau )$, so that one can obtain a time-dependent MAI family ${{\mathcal{A}}}_{\rm{MAI}}(\tau )=({{\bf{X}}}_{\rm{MAI}}^{(1)}(\tau ),\cdots \,,{{\bf{X}}}_{\rm{MAI}}^{(M)}(\tau ))$. With these accessible measurements settings at hand, the vectors for phase generator and measurements can be reexpressed as ${\bf{G}}=R{{\mathcal{A}}}_{\rm{L}}$ and ${\bf{X}}(\tau )=S{{\mathcal{A}}}_{\rm{MAI}}(\tau )$, where *R* and *S* are *M* × (*MK*) real matrices satisfying $R{R}^{T}=S{S}^{T}={{\mathbb{I}}}_{M}$.

According to the method proposed in ref. ^4^, for any transformation matrix *R*, the maximum moment matrix in Eq. (2) is given by $\mathop{\max}\limits_{{\bf{X}}\in \rm{span}({{\mathcal{A}}}_{\rm{MAI}}(\tau))}{\bf{M}}[\rho ,{\bf{G}},{\bf{X}}(\tau)]=R{\bf{M}}[\rho ,{{\mathcal{A}}}_{\rm{L}},{{\mathcal{A}}}_{\rm{MAI}}(\tau)]{R}^{T}$, where ${\bf{M}}[\rho ,{{\mathcal{A}}}_{{\rm{L}}},{{\mathcal{A}}}_{{\rm{M}}{\rm{A}}{\rm{I}}}(\tau)]={\bf{C}}{[\rho ,{{\mathcal{A}}}_{{\rm{L}}},{{\mathcal{A}}}_{{\rm{M}}{\rm{A}}{\rm{I}}}(\tau )]}^{T}\,{\mathbf{\Gamma}}{[\rho ,{{\mathcal{A}}}_{{\rm{M}}{\rm{A}}{\rm{I}}}(\tau)]}^{-1}{\bf{C}}[\rho ,{{\mathcal{A}}}_{{\rm{L}}},{{\mathcal{A}}}_{{\rm{M}}{\rm{A}}{\rm{I}}}(\tau)]$ is the moment matrix (see derivations in Methods). Based on the moment matrix, one can obtain the optimal squeezing matrix in Eq. (3)

6 $$\begin{array}{l}{{\boldsymbol{\Xi }}}_{\rm{opt}}^{2}[\rho ,{\bf{G}},{{\mathcal{A}}}_{\rm{MAI}}(\tau )]:=\mathop{\min }\limits_{{\bf{X}}(\tau )\in \rm{span}({{\mathcal{A}}}_{\rm{MAI}}(\tau ))}{{\boldsymbol{\Xi }}}^{2}[\rho ,{\bf{G}},{\bf{X}}(\tau )]\\ ={{\bf{F}}}_{\rm{SN}}^{\frac{1}{2}}[{\bf{G}}]R{\bf{M}}{[\rho ,{{\mathcal{A}}}_{\rm{L}},{{\mathcal{A}}}_{\rm{MAI}}(\tau )]}^{-1}{R}^{T}{{\bf{F}}}_{\rm{SN}}^{\frac{1}{2}}[{\bf{G}}],\end{array}$$

thus the covariance matrix in Eq. (1) can be reexpressed as $\Sigma [\rho ,{\bf{G}},{{\mathcal{A}}}_{\rm{MAI}}(\tau )]={\Sigma }_{\rm{SN}}^{\frac{1}{2}}{{\boldsymbol{\Xi }}}_{\rm{opt}}^{2}[\rho ,{\bf{G}},{{\mathcal{A}}}_{\rm{MAI}}(\tau )]{\Sigma }_{\rm{SN}}^{\frac{1}{2}}$. For a given vector **n** and an accessible time range *T* for the MAI unitary evolution, the relative estimation uncertainty of a linear combination of imprinted parameters is given by

7 $${\xi }^{-2}({\bf{n}})=\mathop{\max }\limits_{\tau \in \rm{span}(T)}\frac{{{\bf{n}}}^{T}{\Sigma }_{\rm{SN}}{\bf{n}}}{{{\bf{n}}}^{T}\Sigma [\rho ,{\bf{G}},{{\mathcal{A}}}_{\rm{MAI}}(\tau )]{\bf{n}}}.$$

Here, *ξ*^−2^(**n**) > 1 reveals metrologically useful squeezing.

### Multiparameter squeezing for collective spin system

Let us first benchmark MAI strategies with a paradigmatic case of spin squeezed states in atomic experiments. These states are of immediate practical relevance for atomic ensembles, and have been widely used in quantum metrology to overcome the classical shot-noise limit. Moreover, state-of-the-art atomic experiments^16,34–36^ have demonstrated that the entanglement among the constituent particles can be preserved even after spin-squeezed states are spatially split into different spatial modes. Such distributed entanglement is found to enable uncertainty reduction in multi-parameter estimation^4,17^, making split spin-squeezed states a promising resource for distributed quantum sensing.

We consider the scenario where an ensemble of *N* spin-1/2 particles is initially prepared in coherent spin states $| {\psi }_{0}\rangle$ polarized along the *x* direction. Then, the spin states evolve for a time *t* according to the one-axis twisting (OAT) interaction described by the nonlocal Hamiltonian

8 $${\widetilde{H}}_{\rm{nl}}=\hslash \chi \left(\mathop{\sum }\limits_{m=1}^{M}{S}_{z}^{(m)}\right)^{2}.$$

Here, *M* is the mode number and *χ* is the interaction strength. ${S}_{z}^{(m)}={\sum }_{i}^{{N}_{m}}{\sigma }_{z}^{(i)}/2$ represents the collective spin operators for the *N*_*m*_ atoms in the *m*-th mode. This nonlocal Hamiltonian yields the mode entangled states ${\rho }_{\rm{ME}}=| {\psi }_{\rm{ME}}\rangle \langle {\psi }_{\rm{ME}}|$, $| {\psi }_{\rm{ME}}\rangle ={e}^{-i{\widetilde{H}}_{\rm{nl}}t/\hslash }| {\psi }_{0}\rangle$, while simultaneously generating entanglement across the *M* nodes and among all atoms, denoted as mode entanglement and particle entanglement, respectively^17,37,38^. To understand the metrological advantage resulting from these different types of entanglement, we take as reference the state prepared through the local Hamiltonian

9 $${\widetilde{H}}_{\rm{loc}}=\hslash \chi \mathop{\sum }\limits_{m=1}^{M}\left({S}_{z}^{(m)}\right)^{2}.$$

This operator can generate particle entanglement locally, although it is incapable of producing mode entanglement. The resulting states are mode separable states ${\rho }_{\rm{MS}}=| {\psi }_{\rm{MS}}\rangle \langle {\psi }_{\rm{MS}}|$, $| {\psi }_{\rm{MS}}\rangle ={e}^{-i{\widetilde{H}}_{\rm{loc}}t/\hslash }| {\psi }_{0}\rangle$. After state preparation, the phase generator ${\bf{G}}=({S}_{{{\boldsymbol{g}}}_{1}}^{(1)},\cdots \,,{S}_{{{\boldsymbol{g}}}_{M}}^{(M)})$ imprints a vector of unknown phases (*θ*_1_, ⋯ , *θ*_*M*_) to the probe through ${e}^{-i{\sum }_{m}{S}_{{{\boldsymbol{g}}}_{m}}^{(m)}{\theta }_{m}}\rho {e}^{i{\sum }_{m}{S}_{{{\boldsymbol{g}}}_{m}}^{(m)}{\theta }_{m}}$, where ${S}_{{{\boldsymbol{g}}}_{m}}^{(m)}$ denotes a local collective spin observable along ***g***_*m*_ direction on mode *m*.

In typical strategies considering linear measurement observables, the probe states are directly detected without additional evolution, i.e., $U={\mathbb{I}}$. Since both squeezing and anti-squeezing directions are on the *yz*-plane, one can optimize the generator **G** and measurement **X** directions over the basis ${{\mathcal{L}}}^{(m)}=({S}_{y}^{(m)},{S}_{z}^{(m)})$. In MAI strategies, on the other hand, an additional evolution $U(\tau )={e}^{-i\frac{H}{\hslash }\tau }$ before linear measurements is considered. Naturally, we choose the MAI Hamiltonian *H* to be of the same type of the state-preparation Hamiltonian $\widetilde{H}$, so that it will map the final state to an approximately Gaussian state^28^. To further simplify our calculations, we first focus on the simplest task of estimating equal-weight linear combination of phases. The combination can be expressed in terms of equal weights and arbitrary signs, i.e., $\Theta ={{\bf{n}}}^{T}{\boldsymbol{\theta }}={\sum }_{m=1}^{M}{n}_{m}{\theta }_{m}$ with ${n}_{m}=\pm 1/\sqrt{M}$. Since commuting phase generators are considered, the maximum sensitivity after measurement optimization is independent of the signs ( ± ) in **n**, that is *ξ*^−2^(**n**) = *ξ*^−2^. In this symmetric case, we also consider the local atom number and local MAI evolutions in each sub-ensemble to be the same, i.e., *N*_*m*_ = *N*/*M* and ${U}_{\rm{loc}}^{(m)}({\tau }^{(m)})={U}_{\rm{loc}}(\tau )$. We can then construct the families of MAI measurements as ${{\bf{X}}}_{\rm{MAI},\alpha }^{(m)}=({U}_{\alpha }^{\dagger }(\tau ){S}_{y}^{(m)}{U}_{\alpha }(\tau ),{U}_{\alpha }^{\dagger }(\tau ){S}_{z}^{(m)}{U}_{\alpha }(\tau ))$, where ${U}_{\rm{loc}}(\tau )={e}^{-i\chi \tau {({S}_{z}^{(m)})}^{2}}$ and ${U}_{\rm{nl}}(\tau )={e}^{-i\chi \tau {({\sum }_{m}{S}_{z}^{(m)})}^{2}}$ are the unitary operators in the local and nonlocal MAI strategies, respectively. In Supplementary Note 1. C, we extend the analysis to arbitrarily weighted parameter combinations, and emphasize that in this general setting the optimal strategy of particle distribution depends on the specific linear combination.

**Fig. 2:**
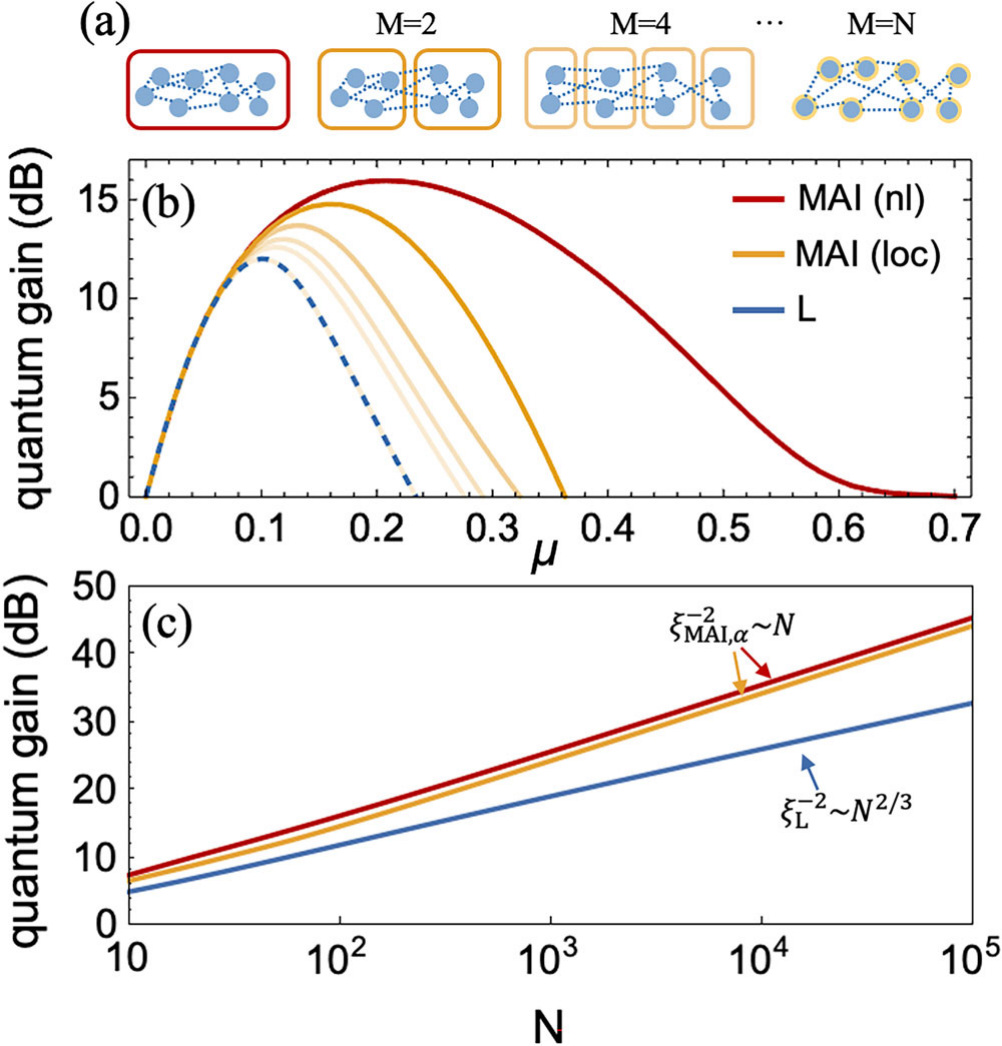
Multiparameter squeezing for mode-entangled spin squeezed states *ρ*_ME_ under different measurement strategies. **a** Illustration of nonlocal and local MAI: The nonlocal MAI is performed on the entire ensemble of *N* particles (red box), while the local MAI are separately performed on *N*_*m*_ particles within each modes (yellow boxes). The number of local atoms *N*_*m*_ will decrease as *M* increases, and eventually reaches *N*_*m*_ = 1 when *M* = *N*. **b** Quantum gain $10{\log }_{10}({\xi }^{-2})$ as a function of the evolution time *μ* = 2*χt*, for *N* = 100. The sensitivities detected from nonlocal MAI (red solid line) and direct linear measurements without any additional evolution (blue solid line) are independent of *M*. The sensitivity from local MAI will decrease with *M* (yellow lines), where the opacity decreases with mode number *M* (*M* = 2, 4, 10, 20, 100 is shown). **c** Sensitivity scaling for linear measurements, nonlocal MAI (independent with *M*) and local MAI (*M* = 2) as the function of total atom number *N*. Both MAI protocols yield Heisenberg scaling ${\xi }_{\rm{MAI},\alpha }^{-2} \sim N$, which significantly outperform the one from linear measurement ${\xi }_{\rm{L}}^{-2} \sim {N}^{2/3}$.

We present in Fig. 2b the multiparameter sensitivity for mode-entangled spin-squeezed states *ρ*_ME_ as a function of OAT evolution time *μ* = 2*χt*. At short evolution times, OAT spin states are nearly Gaussian, thus both linear and MAI strategies can detect their metrological usefulness. However, as *μ* increases the states will become over-squeezed and thus non-Gaussian. A quantitative analysis of its non-Gaussian properties during OAT dynamics is presented in Supplementary Note 2. In such non-Gaussian regime the sensitivity revealed by linear measurements decay fast. MAI strategies, on the contrary, keep showing quantum-enhanced sensitivity for a wider time-evolution range, which is due to the additional unitary before measurement. Based on the analytical expressions of moment matrices in Supplementary Note 1, we find that the resulting sensitivity of nonlocal MAI and linear measurements is independent with mode number *M*. However, this is not the case for local MAI. When *M* is small, local MAI strategy can achieve a sensitivity comparable to the nonlocal MAI strategy. While its sensitivity decreases with increasing *M*, and eventually matches that of linear measurements in the extreme case of *M* = *N*, where the OAT Hamiltonian acts trivially for individual spin-1/2 particles. The reason is that local MAI solely acts on *N*_*m*_ particles within each mode, is therefore insensitive to entanglement across different modes. As *M* increases and *N*_*m*_ decreases, the amount of mode entanglement left unexploited by local MAI grows, leading to a reduced metrological gain. In addition, we compare the multiparameter sensitivity for mode separable states *ρ*_MS_ in Supplementary Note 1, to verify the contribution of mode entanglement to the metrological advantage^4,8,17^.

The scaling laws of multiparameter sensitivity for *ρ*_ME_ as a function of total atom number *N* are shown in Fig. 2c, and the details of analytical solutions for associated moment matrix are given in Supplementary Note 1. For the experimentally practical case of short times scales and relatively large atom number *N*, we observe that the multiparameter sensitivity for the nonlocal MAI protocol can reach the Heisenberg scaling ${\xi }_{\rm{MAI},\rm{nl}}^{-2} \sim N$ (red line), under the optimal evolution times *t*_*opt*_ ≈ *τ*_*opt*_ ~ *N*^−1/2^. The sensitivity scaling from linear measurements is ${\xi }_{\rm{L}}^{-2} \sim {N}^{2/3}$ (blue line), with the optimal times *t*_opt_ ~ *N*^−2/3^. Therefore, a significant advantage of nonlocal MAI strategy is shown. In Supplementary Note 1, we prove that although the moment matrices in both linear measurements and nonlocal MAI strategies depend on the mode number *M*, the maximum multiparameter squeezing coefficients achieved by either strategy are independent of *M*. Furthermore, in Fig. 2c we also plot the sensitivity scaling for the local MAI protocol in the specific case of *M* = 2 (yellow line). Under the optimal MAI evolution times *τ*_opt_ ≈ 2*t*, the local MAI strategy exhibits a metrological advantage comparable to the nonlocal MAI, and is also able to reach the Heisenberg scaling ${\xi }_{\rm{MAI},\rm{loc}}^{-2} \sim N$.

### Multiparameter squeezing for continuous-variable system

As a simple, yet immediately practical, example in continuous-variable (CV) systems, we investigate the advantage of implementing the local/nonlocal MAI strategies on a two-mode squeezed vacuum (TMSV) state. Such states are routinely prepared in various CV platforms, and allow for quantum-enhanced (multi)parameter estimation tasks in SU(1,1)-type interferometers^39^. Mathematically, the TMSV state is obtained by applying the two-mode squeezing operator ${S}_{AB}=\exp [-\zeta {a}^{\dagger }{b}^{\dagger }+{\zeta }^{* }ab]$ to the vacuum, $| \rm{TMSV}\rangle ={S}_{AB}| 0\rangle$, where *ζ* = *re*^*iϕ*^ is the squeezing parameter. Two parameters are encoded via a displacement operations, ${e}^{-i({G}_{1}{d}_{1}+{G}_{2}{d}_{2})}| \rm{TMSV}\rangle$, where the local displacement amplitudes *d*_*m*_ can be effectively regarded as *θ*_*m*_. The family of linear measurements contains quadrature operators ${\mathcal{L}}=(x,p)$ with $x=(a+{a}^{\dagger })/\sqrt{2},p=-i(a-{a}^{\dagger })/\sqrt{2}$. Then, the additional evolutions in nonlocal and local MAI strategies can be considered as a two-mode squeezing operator ${U}_{\rm{nl}}=\exp [{\zeta }_{\rm{nl}}{a}^{\dagger }{b}^{\dagger }-{\zeta }_{\rm{nl}}^{* }ab]$ and one-mode squeezing operators ${U}_{{\mathrm{loc}}}=\exp [({\zeta }_{{\mathrm{loc}}}{({a}^{\dagger })}^{2}-{\zeta }_{{\mathrm{loc}}}^{* }{a}^{2})/2]$ with ${\zeta }_{\alpha }={r}_{\alpha }{e}^{i{\phi }_{\alpha }}$, respectively. In a multimode displacements estimation task with Gaussian states, the linear measurements are optimal to saturate quantum Cram$\acute{e}$r-Rao bound. Therefore, we find that the linear measurements and MAI strategies will yield the same sensitivity, ${\xi }_{\rm{L}}^{-2}={\xi }_{\rm{MAI},\alpha }^{-2}={e}^{2r}$, which indicates the reveal of metrological entanglement if *r* > 0 (see Supplementary Note 3. A). In such ideal scenario, the sensitivity is independent of MAI squeezing *r*_*α*_, indicating that the MAI schemes do not offer any enhancement in metrological advantage.

**Fig. 3:**
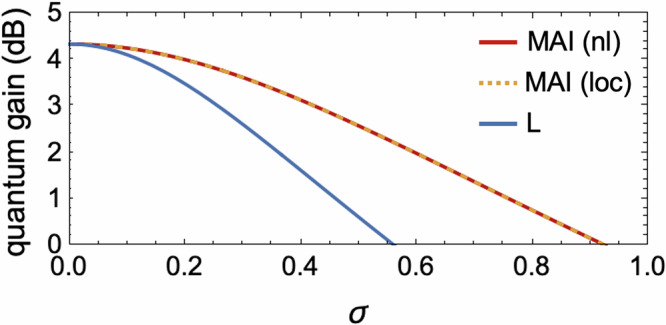
Detection noise effects on the multiparameter sensitivity. For a two-mode squeezed vacuum state with squeezing parameter *r* = 0.5, we show the quantum gain obtained from linear measurements and MAI strategies (*r*_nl_ = *r*_loc_ = *r*) as a function of standard derivation *σ* of Gaussian-distributed detection noise.

However, when the effects of detection noise are taken into account, we show that MAI schemes will exhibit enhanced noise robustness. Consider Gaussian-distributed detection noises with standard deviation *σ* (see Supplementary Note 3. A), the ratio of the sensitivities from typical and MAI strategies is given as

10 $$\frac{{\xi }_{\rm{L}}^{-2}}{{\xi }_{\rm{MAI},\alpha }^{-2}}=\frac{{e}^{-2r}+2{\sigma }^{2}{e}^{-2{r}_{\alpha }}}{{e}^{-2r}+2{\sigma }^{2}},$$

demonstrating that MAI strategies can outperform linear measurements if *r*_*α*_ > 0. In Fig. 3, we present the quantum gain as a function of squeezing level *r*, for *r*_loc_ = *r*_nl_ = *r*. Both MAI schemes show significant advantage on noise robustness over the typical scheme.

In realistic implementations, highly squeezed states generally carry large excitation energies. Once these go beyond the linear regime of the experimental platform, the inevitable nonlinearity drives the states into non-Gaussian states. In Supplementary Note 3. B, we investigate the probe states generated by the interplay of squeezing and Kerr nonlinearity, and show that MAI strategies yield higher sensitivities for non-Gaussian CV states than linear measurements.

## Discussions

We have characterized the performances of MAI strategies in distributed quantum sensing, showing in which cases they allow for an enhancement in multiparameter sensitivity. In the considered MAI strategies, an additional evolution, which can be either nonlocal or local, is implemented before a linear measurement. Benchmarking with both discrete- and continuous-variable systems, we show that MAI strategies significantly outperform the typical scenario in detection of non-Gaussian sensitivity as well as robustness against detection noise, without any change in the amount of detection statistics to be collected. Furthermore, we give the analytical solutions for multiparameter squeezing for the spin states generated by the one-axis-twisting (OAT) interaction, and reveal the fundamental principles of multiparameter estimation under different measurement strategies. Surprisingly, we show that the Heisenberg scalings can be achieved in both MAI protocols.

In our framework of MAI strategies, the evolution is not limited to specific Hamiltonians. If a wider class of experimentally accessible interactions is available, the additional evolution can be further designed and optimized, e.g., by using reinforcement learning^40^, to reach a higher sensitivity. Similar ideas could be explored to use MAI strategies for the detection of non-Gaussian quantum correlations^41^. Besides providing timely and powerful tools in multiparameter estimation scenarios, our work expands the metrological applications of non-Gaussian states, and provide practical guidelines for implementing distributed sensing tasks in the state-of-the-art experiments.

## Methods

### Methods of moment

We use the methods of moment in ref. ^4^ to analytically optimize the measurements among a set of accessible operators in the MAI protocols. Let us first consider a family of linear measurements ${{\mathcal{A}}}_{\rm{L}}=({{\mathcal{L}}}^{(1)},\cdots \,,{{\mathcal{L}}}^{(M)})$, where ${{\mathcal{L}}}^{(m)}=({L}_{1}^{(m)},\cdots \,,{L}_{K}^{(m)})$ represent the vectors of accessible linear operators on the *m*-th mode. These linear measurements include collective spin observables in discrete-variable systems and linear quadrature (i.e., homodyne) measurements in continuous-variable systems. In linear-encoding scenarios, a local phase generator can be expressed by ${G}_{m}={{\bf{g}}}_{m}^{T}{{\mathcal{L}}}^{(m)}={\sum }_{k=1}^{K}{g}_{m,k}{L}_{k}^{(m)}$, such that the family consisting of *M* commuting generators is given as ${\bf{G}}=({G}_{1},\cdots \,,{G}_{M})=R{{\mathcal{A}}}_{\rm{L}}$, where

11 $$R=\left(\begin{array}{rcl}{{\bf{g}}}_{1} & \cdots & {\bf{0}}\\ \vdots & \ddots & \vdots \\ {\bf{0}} & \cdots & {{\bf{g}}}_{M}\end{array}\right)$$

is an *M* × (*MK*) real matrix satisfying $R{R}^{T}={{\mathbb{I}}}_{M}$. Then, given the unitary evolutions for MAI technique, ${U}_{\alpha }={e}^{-i\frac{{H}_{\alpha }}{\hslash }\tau }$ with *α* = {nl, loc}, one can construct the vectors of measurements on *m*-th mode, ${{\bf{X}}}_{\rm{MAI},\alpha }^{(m)}(\tau )=({U}_{\alpha }^{\dagger }(\tau ){L}_{1}^{(m)}{U}_{\alpha }(\tau ),\cdots \,,{U}_{\alpha }^{\dagger }(\tau ){L}_{K}^{(m)}{U}_{\alpha }(\tau ))$, and further obtain a time-dependent MAI vector on the entire *M*-mode system ${{\mathcal{A}}}_{\rm{MAI}}(\tau )=({{\bf{X}}}_{\rm{MAI}}^{(1)}(\tau ),\cdots \,,{{\bf{X}}}_{\rm{MAI}}^{(M)}(\tau ))$. Based on such family, any measurements can thus be given as ${\bf{X}}(\tau )=S{{\mathcal{A}}}_{\rm{MAI}}(\tau )$, where *S* is an *M* × (*MK*) real matrix satisfying $S{S}^{T}={{\mathbb{I}}}_{M}$.

Using ${\bf{C}}[\rho ,{\bf{G}},{\bf{X}}(\tau )]=S{\bf{C}}[\rho ,{{\mathcal{A}}}_{\rm{L}},{{\mathcal{A}}}_{\rm{MAI}}(\tau )]{R}^{T}$ and ${\mathbf{\Gamma }}[\rho ,{\bf{X}}(\tau )]=S{\mathbf{\Gamma }}[\rho ,{{\mathcal{A}}}_{{\rm{M}}{\rm{A}}{\rm{I}}}(\tau )]{S}^{T}$, the moment matrix in Eq. (2) can be reexpressed as

12 $$\begin{array}{l}{\bf{M}}[\rho ,{\bf{G}},{\bf{X}}(\tau )]:={\bf{C}}{[\rho ,{\bf{G}},{\bf{X}}(\tau )]}^{T}{\mathbf{\Gamma }}{[\rho ,{\bf{X}}(\tau )]}^{-1}{\bf{C}}[\rho ,{\bf{G}},{\bf{X}}(\tau )]\\ =R{\bf{C}}{[\rho ,{{\mathcal{A}}}_{{\rm{L}}},{{\mathcal{A}}}_{{\rm{M}}{\rm{A}}{\rm{I}}}(\tau )]}^{T}{S}^{T}{(S{\mathbf{\Gamma }}[\rho ,{{\mathcal{A}}}_{{\rm{M}}{\rm{A}}{\rm{I}}}(\tau )]{S}^{T})}^{-1}S\\ \cdot {\bf{C}}[\rho ,{{\mathcal{A}}}_{{\rm{L}}},{{\mathcal{A}}}_{{\rm{M}}{\rm{A}}{\rm{I}}}(\tau )]{R}^{T}\\ \le R{\bf{M}}[\rho ,{{\mathcal{A}}}_{{\rm{L}}},{{\mathcal{A}}}_{{\rm{M}}{\rm{A}}{\rm{I}}}(\tau )]{R}^{T},\end{array}$$

where we define the moment matrix ${\bf{M}}[\rho ,{{\mathcal{A}}}_{{\rm{L}}},{{\mathcal{A}}}_{{\rm{M}}{\rm{A}}{\rm{I}}}(\tau )]={\bf{C}}{[\rho ,{{\mathcal{A}}}_{{\rm{L}}},{{\mathcal{A}}}_{{\rm{M}}{\rm{A}}{\rm{I}}}(\tau )]}^{T}{\mathbf{\Gamma }}{[\rho ,{{\mathcal{A}}}_{{\rm{M}}{\rm{A}}{\rm{I}}}(\tau )]}^{-1}{\bf{C}}[\rho ,{{\mathcal{A}}}_{{\rm{L}}},{{\mathcal{A}}}_{{\rm{M}}{\rm{A}}{\rm{I}}}(\tau )]$. Here the inequality in the last line derived from the matrix-valued Cauchy-Schwarz inequality^4^. It is saturated if the measurement **X**(*τ*) is optimized over the accessible vector ${{\mathcal{A}}}_{\rm{MAI}}(\tau )$, i.e., $\mathop{\max }\limits_{{\bf{X}}(\tau )\in \rm{span}({{\mathcal{A}}}_{\rm{MAI}}(\tau ))}{\bf{M}}[\rho ,{\bf{G}},{\bf{X}}(\tau )]=R{\bf{M}}[\rho ,{{\mathcal{A}}}_{\rm{L}},{{\mathcal{A}}}_{\rm{MAI}}(\tau )]{R}^{T}$. For any given generator matrix *R*, the matrix *S* corresponding to optimal measurements is given as $GS=R{\bf{C}}{[\rho ,{{\mathcal{A}}}_{{\rm{L}}},{{\mathcal{A}}}_{{\rm{M}}{\rm{A}}{\rm{I}}}(\tau )]}^{T}{\mathbf{\Gamma }}{[\rho ,{{\mathcal{A}}}_{{\rm{M}}{\rm{A}}{\rm{I}}}(\tau )]}^{-1}$, with a real matrix *G* for normalization.

For a give generator vector **G**, the squeezing matrix optimizing over accessible measurements ${{\mathcal{A}}}_{\rm{MAI}}(\tau )$ can be written in terms of the moment matrix,

13 $$\begin{array}{l}{\Xi }_{\rm{opt}}^{2}[\rho ,{\bf{G}},{{\mathcal{A}}}_{\rm{MAI}}(\tau )]=\mathop{\min }\limits_{{\bf{X}}(\tau )\in \rm{span}({{\mathcal{A}}}_{\rm{MAI}}(\tau ))}{\Xi }^{2}[\rho ,{\bf{G}},{\bf{X}}(\tau )]\\ =\mathop{\min }\limits_{{\bf{X}}(\tau )\in \rm{span}({{\mathcal{A}}}_{\rm{MAI}}(\tau ))}{{\bf{F}}}_{\rm{SN}}{[{\bf{G}}]}^{\frac{1}{2}}{\bf{M}}{[\rho ,{\bf{G}},{\bf{X}}]}^{-1}{{\bf{F}}}_{\rm{SN}}{[{\bf{G}}]}^{\frac{1}{2}}\\ ={{\bf{F}}}_{\rm{SN}}{[{\bf{G}}]}^{\frac{1}{2}}R{\bf{M}}{[\rho ,{{\mathcal{A}}}_{\rm{L}},{{\mathcal{A}}}_{\rm{MAI}}(\tau )]}^{-1}{R}^{T}{{\bf{F}}}_{\rm{SN}}{[{\bf{G}}]}^{\frac{1}{2}},\end{array}$$

which yields the corresponding covariance matrix

14 $$\Sigma [\rho ,{\bf{G}},{{\mathcal{A}}}_{\rm{MAI}}(\tau )]={\Sigma }_{\rm{SN}}^{\frac{1}{2}}{\Xi }_{\rm{opt}}^{2}[\rho ,{\bf{G}},{{\mathcal{A}}}_{\rm{MAI}}(\tau )]{\Sigma }_{\rm{SN}}^{\frac{1}{2}}.$$

After optimizing *R* and the evolution times *τ*, the covariance matrix can be further optimized as $\Sigma =\mathop{\min }\limits_{R,\tau }\Sigma [\rho ,{\bf{G}},{{\mathcal{A}}}_{\rm{MAI}}(\tau )]$. For a given linear combination **n**, one can finally obtain the relative estimation uncertainty as

15 $${\xi }^{-2}({\bf{n}})=\frac{{{\bf{n}}}^{T}{\Sigma }_{\rm{SN}}{\bf{n}}}{{{\bf{n}}}^{T}\Sigma {\bf{n}}}.$$

We assume the vector ${\bf{n}}=({n}_{1},\cdots \,,{n}_{M})$ containing equally distributed elements with different signs, i.e., ${n}_{m}=\pm \frac{1}{\sqrt{M}}$. In our scenarios where the phase generators are locally implemented, we find that the resulting multiparameter sensitivity is independent with the array of signs: *ξ*^−2^(**n**) = *ξ*^−2^.

### Measurement optimization in spin ensembles

In the atomic ensembles we considered, since the squeezing of the OAT spin states mostly lie in the *yz* − plane, the vector of linear operators on each mode is set as ${{\mathcal{L}}}^{(m)}=({S}_{y}^{(m)},{S}_{z}^{(m)})$, where ${S}_{\beta }^{(m)}={\sum }_{i}^{{N}_{m}}{\sigma }_{\beta }^{(i)}/2$ is local collective spin observables for *N*_*m*_ atoms in the *m*-th mode. The additional state evolution in nonlocal and local MAI protocols can be described by the unitary operator ${U}_{\rm{nl}}(\tau )={e}^{-i\chi \tau {({\sum }_{m=1}^{M}{S}_{z}^{(m)})}^{2}}$ and ${U}_{{\mathrm{loc}}}^{(m)}(\tau )={e}^{-i\chi \tau {({S}_{z}^{(m)})}^{2}}$, respectively, which yields the corresponding vectors ${{\bf{X}}}_{\rm{MAI},\alpha }^{(m)}=({U}_{\alpha }^{\dagger }(\tau ){S}_{y}^{(m)}{U}_{\alpha }(\tau ),{U}_{\alpha }^{\dagger }(\tau ){S}_{z}^{(m)}{U}_{\alpha }(\tau ))$. Then, based on ${{\mathcal{L}}}^{(m)}$ and ${{\bf{X}}}_{\rm{MAI},\alpha }^{(m)}$, we can obtain the family of accessible linear and MAI measurements on *M*-mode ${{\mathcal{A}}}_{\rm{L}}$ and ${{\mathcal{A}}}_{\rm{MAI},\alpha }$.

To simplify the model, the atom numbers in every sub-ensembles are assumed to be the same, i.e., *N*_*m*_ = *N*/*M*. In this case, the 2*M* × 2*M* covariance matrix can be expressed in terms of 2 submatrices **Γ**_*mm*_ and **Γ**_*mn*_,

16 $${\mathbf{\Gamma }}=\left(\begin{array}{cccc}{{\mathbf{\Gamma }}}_{mm} & {{\mathbf{\Gamma }}}_{mn} & \cdots & {{\mathbf{\Gamma }}}_{mn}\\ {{\mathbf{\Gamma }}}_{mn} & {{\mathbf{\Gamma }}}_{mm} & \cdots & {{\mathbf{\Gamma }}}_{mn}\\ \vdots & \vdots & \ddots & \vdots \\ {{\mathbf{\Gamma }}}_{mn} & {{\mathbf{\Gamma }}}_{mn} & \cdots & {{\mathbf{\Gamma }}}_{mm}\end{array}\right),$$

where **Γ**_*mm*_ is a 2 × 2 covariance matrix of local operators, whose elements are ${({\Gamma }_{mm})}_{ij}=\rm{Cov}({{\mathcal{A}}}_{\rm{MAI},\alpha ,i}^{(m)},{{\mathcal{A}}}_{\rm{MAI},\alpha ,j}^{(m)})$, **Γ**_*mn*_ is a 2 × 2 covariance matrix of nonlocal operators, with elements ${({\Gamma }_{mn})}_{ij}=\rm{Cov}({{\mathcal{A}}}_{\rm{MAI},\alpha ,i}^{(m)},{{\mathcal{A}}}_{\rm{MAI},\alpha ,j}^{(n)})(m\ne n)$. For mode separable pure states *ρ*_MS_, the submatrices of nonlocal operators become zero matrix **Γ**_*mn*_ = **0**_2×2_, thus the covariance matrix can be expressed as ${\mathbf{\Gamma }}={\oplus }_{m=1}^{M}{{\mathbf{\Gamma }}}_{mm}$.

Similarly, the 2*M* × 2*M* commutator matrix ***C*** can be expressed in terms of submatrices ***C***_*mm*_ and ***C***_*mn*_

17 $${\boldsymbol{C}}=\left(\begin{array}{cccc}{{\boldsymbol{C}}}_{mm} & {{\boldsymbol{C}}}_{mn} & \cdots & {{\boldsymbol{C}}}_{mn}\\ {{\boldsymbol{C}}}_{mn} & {{\boldsymbol{C}}}_{mm} & \cdots & {{\boldsymbol{C}}}_{mn}\\ \vdots & \vdots & \ddots & \vdots \\ {{\boldsymbol{C}}}_{mn} & {{\boldsymbol{C}}}_{mn} & \cdots & {{\boldsymbol{C}}}_{mm}\end{array}\right),$$

where ***C***_*mm*_ is a 2 × 2 commutator matrix of local operators, with elements ${({C}_{mm})}_{ij}=i\langle [{{\mathcal{L}}}_{i}^{(m)},{{\bf{X}}}_{\rm{MAI},\alpha ,j}^{(m)}]\rangle$, and ***C***_*mn*_ is a 2 × 2 commutator matrix of nonlocal operators, with elements ${({C}_{mn})}_{ij}=i\langle [{{\mathcal{L}}}_{i}^{(m)},{{\bf{X}}}_{\rm{MAI},\alpha ,j}^{(n)}]\rangle$. In the local MAI protocols, the submatrices ***C***_*mn*_ = **0**_2×2_ will lead to ${\boldsymbol{C}}={\oplus }_{m=1}^{M}{{\boldsymbol{C}}}_{mm}$. Based on **Γ** and ***C***, the moment matrix **M** = ***C***^*T*^**Γ*C*** can be constructed.

To optimize the phase generators matrix *R*, we express the local vectors **g**_*m*_ in terms of measurement angles *ϕ*_*m*_: ${{\bf{g}}}_{m}=(\cos ({\phi }_{m}),\sin ({\phi }_{m}))$. Therefore, the minimized squeezing matrix is

18 $$\begin{array}{l}\min \, \mathop{\Xi }^{2}_{\mathrm{opt}}[\rho ,{\bf{G}},{{\mathcal{A}}}_{\mathrm{MAI}}(\tau )]\\ =\mathop{\min }\limits_{\tau \in \mathrm{span}T}\mathop{\min }\limits_{{\phi }_{1},\cdots \,,{\phi }_{M}}{{\bf{F}}}_{\mathrm{SN}}{[{\bf{G}}]}^{\frac{1}{2}}R{\bf{M}}{[\rho ,{{\mathcal{A}}}_{{\rm{L}}},{{\mathcal{A}}}_{\mathrm{MAI}}(\tau )]}^{-1}{R}^{T}{{\bf{F}}}_{\mathrm{SN}}{[{\bf{G}}]}^{\frac{1}{2}},\end{array}$$

where *T* is accessible time range and ${{\bf{F}}}_{\rm{SN}}=diag({N}_{1},\cdots \,,{N}_{M})$. Because the metrological sensitivity is independent with the arrays of signs in **n**, we can set ${\bf{n}}={{\bf{n}}}_{+}=\frac{1}{\sqrt{M}}(1,\cdots \,,1)$ without loss of generality, in which case the optimal local directions for *R* are the same, i.e., *ϕ*_*m*_ = *ϕ*.

## Supplementary information

Supplementary Information

## Data Availability

The data that supports the findings of this study are available on Zenodo at 10.5281/zenodo.18508841.

## Code availability

The code that supports the findings of this study are available on Zenodo at 10.5281/zenodo.18508841.
