# Supplementary material for: Distributed quantum sensing with measurement-after-interaction strategies
Source: npj Quantum Inf. 2026 Apr 3;12(1):84. doi: 10.1038/s41534-026-01224-z (PMC13218934; doi:10.1038/s41534-026-01224-z)
Supplement: Supplementary file 1 — Supplementary Information [file 41534_2026_1224_MOESM1_ESM.pdf]

Supplementary material for "Distributed quantum sensing with measurement-after-interaction strategies"

Jiajie Guo,[1] Shuheng Liu,[1] Matteo Fadel,[2,*] and Qiongyi He[1,3,4,5,†]

[1]State Key Laboratory for Mesoscopic Physics, School of Physics, Frontiers Science Center for Nano-optoelectronics, & Collaborative Innovation Center of Quantum Matter, Peking University, Beijing 100871, China
[2]Department of Physics, ETH Zürich, 8093 Zürich, Switzerland
[3]Collaborative Innovation Center of Extreme Optics, Shanxi University, Taiyuan, Shanxi 030006, China
[4]Peking University Yangtze Delta Institute of Optoelectronics, Nantong 226010, Jiangsu, China
[5]Hefei National Laboratory, Hefei 230088, China

SUPPLEMENTARY NOTE 1. ANALYTICAL SOLUTIONS FOR MULTIPARAMETER MOMENT MATRIX FOR SPIN SQUEEZED STATES

A. Equal-weight combination estimation with local squeezing spin states $\rho_{\rm MS}$

We consider an ensemble of N spin-1/2 particles initially polarized along the x direction are distributed among M spatially-separated modes, and assume the atom number in each mode is the same, i.e. $N_m = N/M = \mathcal{N}$. To generate the local squeezing spin states, the probe state will undergo the OAT interaction described by the local Hamiltonian

$$\tilde{H}_{\rm loc} = \hbar\chi \sum_{m=1}^{M} \left(S_z^{(m)}\right)^2, \tag{1}$$

which can generate the particle entanglement locally in each modes. The resulting mode separable states are written as $\rho_{\rm MS} = |\psi_{\rm MS}\rangle\langle\psi_{\rm MS}|$, where $|\psi_{\rm MS}\rangle = e^{-i\frac{\tilde{H}_{\rm loc}}{\hbar}t}|\psi_0\rangle$. In the following, we denote the OAT squeezing times for state preparation as $\mu = 2\chi t$, and denote the OAT evolution times for MAI strategies as $\mu_\alpha = 2\chi\tau$.

1. *Multiparameter sensitivity for ρ_{MS} in typical protocols using linear measurements*

For mode separable pure states $\rho_{\rm MS}$, the covariance matrix can be expressed by $\mathbf{\Gamma} = \oplus_{m=1}^{M}\mathbf{\Gamma}_{mm}$, where the submatrix $\mathbf{\Gamma}_{mm}$ is given as

$$\mathbf{\Gamma}_{mm} = \begin{pmatrix} {\rm Cov}\left(S_y^{(m)}, S_y^{(m)}\right) & {\rm Cov}\left(S_y^{(m)}, S_z^{(m)}\right) \\ {\rm Cov}\left(S_z^{(m)}, S_y^{(m)}\right) & {\rm Cov}\left(S_z^{(m)}, S_z^{(m)}\right) \end{pmatrix}, \tag{2}$$

$${\rm Cov}\left(S_y^{(m)}, S_y^{(m)}\right) = \frac{1}{8}\mathcal{N}\left(\mathcal{N} + 1 - (\mathcal{N}-1)\cos(\mu)^{\mathcal{N}-2}\right), \tag{3}$$

$${\rm Cov}\left(S_y^{(m)}, S_z^{(m)}\right) = {\rm Cov}\left(S_z^{(m)}, S_y^{(m)}\right) = \frac{1}{4}\mathcal{N}(\mathcal{N}-1)\cos\left(\frac{\mu}{2}\right)^{\mathcal{N}-2}\sin\left(\frac{\mu}{2}\right), \tag{4}$$

$${\rm Cov}\left(S_z^{(m)}, S_z^{(m)}\right) = \frac{\mathcal{N}}{4}. \tag{5}$$

The commutator matrix is $\boldsymbol{C} = \oplus_{m=1}^{M}\boldsymbol{C}_{mm}$, where

$$\boldsymbol{C}_{mm} = i\begin{pmatrix} 0 & \langle[S_y^{(m)}, S_z^{(m)}]\rangle \\ \langle[S_z^{(m)}, S_y^{(m)}] & 0 \end{pmatrix}, \tag{6}$$

$$-i\langle[S_y^{(m)}, S_z^{(m)}]\rangle = i\langle[S_z^{(m)}, S_y^{(m)}]\rangle = \frac{1}{2}\mathcal{N}\cos\left(\frac{\mu}{2}\right)^{\mathcal{N}-1}. \tag{7}$$

* email address: fadelm@phys.ethz.ch
† email address: qiongyihe@pku.edu.cn

The relative estimation uncertainty can be solve as

$$\xi_{\mathrm{L}}^{-2}(\rho_{\mathrm{MS}}) = \frac{4\cos(\mu/2)^{2\frac{N}{M}-2}\cos(\mu)^2}{-\left((-1+\frac{N}{M})\cos(\mu)^{\frac{N}{M}}\right)+\cos(\mu)^2\left(3+\frac{N}{M}-\sqrt{(-1+\frac{N}{M})^2\left(-8\cos(\mu/2)^{2\frac{N}{M}-4}(-1+\cos(\mu))+(\cos(\mu)^2-\cos(\mu)^{\frac{N}{M}})\sec(\mu)^4\right)}\right)}, \tag{8}$$

which is determined by the local atom number in each node N/M.

2. *Multiparameter sensitivity for ρ_{MS} in local MAI protocols*

The $2M\times 2M$ covariance matrix $\mathbf{\Gamma}$ can be written as $\mathbf{\Gamma}=\oplus_{m=1}^{M}\mathbf{\Gamma}_{mm}$, where

$$\mathbf{\Gamma}_{mm} = \begin{pmatrix} \mathrm{Cov}\left(U_{\mathrm{loc}}^\dagger S_y^{(m)}U_{\mathrm{loc}}, U_{\mathrm{loc}}^\dagger S_y^{(m)}U_{\mathrm{loc}}\right) & \mathrm{Cov}\left(U_{\mathrm{loc}}^\dagger S_y^{(m)}U_{\mathrm{loc}}, U_{\mathrm{loc}}^\dagger S_z^{(m)}U_{\mathrm{loc}}\right) \\ \mathrm{Cov}\left(U_{\mathrm{loc}}^\dagger S_z^{(m)}U_{\mathrm{loc}}, U_{\mathrm{loc}}^\dagger S_y^{(m)}U_{\mathrm{loc}}\right) & \mathrm{Cov}\left(U_{\mathrm{loc}}^\dagger S_z^{(m)}U_{\mathrm{loc}}, U_{\mathrm{loc}}^\dagger S_z^{(m)}U_{\mathrm{loc}}\right)\end{pmatrix}, \tag{9}$$

$$\mathrm{Cov}\left(U_{\mathrm{loc}}^\dagger S_y^{(m)}U_{\mathrm{loc}}, U_{\mathrm{loc}}^\dagger S_y^{(m)}U_{\mathrm{loc}}\right) = \frac{1}{8}\mathcal{N}\left(\mathcal{N}+1-(\mathcal{N}-1)\cos(\mu-\mu_{\mathrm{loc}})^{\mathcal{N}-2}\right), \tag{10}$$

$$\mathrm{Cov}\left(U_{\mathrm{loc}}^\dagger S_y^{(m)}U_{\mathrm{loc}}, U_{\mathrm{loc}}^\dagger S_z^{(m)}U_{\mathrm{loc}}\right) = \mathrm{Cov}\left(U_{\mathrm{loc}}^\dagger S_z^{(m)}U_{\mathrm{loc}}, U_{\mathrm{loc}}^\dagger S_y^{(m)}U_{\mathrm{loc}}\right) = \frac{1}{4}\mathcal{N}(\mathcal{N}-1)\cos\left(\frac{\mu-\mu_{\mathrm{loc}}}{2}\right)^{\mathcal{N}-2}\sin\left(\frac{\mu-\mu_{\mathrm{loc}}}{2}\right), \tag{11}$$

$$\mathrm{Cov}\left(U_{\mathrm{loc}}^\dagger S_z^{(m)}U_{\mathrm{loc}}, U_{\mathrm{loc}}^\dagger S_z^{(m)}U_{\mathrm{loc}}\right) = \frac{\mathcal{N}}{4}. \tag{12}$$

The commutator matrix is $\boldsymbol{C}=\oplus_{m=1}^{M}\boldsymbol{C}_{mm}$, where

$$\boldsymbol{C}_{mm} = i\begin{pmatrix} \langle[S_y^{(m)}, U_{\mathrm{loc}}^\dagger S_y^{(m)}U_{\mathrm{loc}}]\rangle & \langle[S_y^{(m)}, U_{\mathrm{loc}}^\dagger S_z^{(m)}U_{\mathrm{loc}}]\rangle \\ \langle[S_z^{(m)}, U_{\mathrm{loc}}^\dagger S_y^{(m)}U_{\mathrm{loc}}]\rangle & \langle[S_z^{(m)}, U_{\mathrm{loc}}^\dagger S_z^{(m)}U_{\mathrm{loc}}]\rangle\end{pmatrix}, \tag{13}$$

$$i\langle[S_y^{(m)}, U_{\mathrm{loc}}^\dagger S_y^{(m)}U_{\mathrm{loc}}]\rangle = -\frac{1}{4}\mathcal{N}(\mathcal{N}-1)\left(\cos\left(\mu-\frac{\mu_{\mathrm{loc}}}{2}\right)^{\mathcal{N}-2}+\cos\left(\frac{\mu_{\mathrm{loc}}}{2}\right)^{\mathcal{N}-2}\right)\sin\left(\frac{\mu_{\mathrm{loc}}}{2}\right), \tag{14}$$

$$i\langle[S_y^{(m)}, U_{\mathrm{loc}}^\dagger S_z^{(m)}U_{\mathrm{loc}}]\rangle = \frac{1}{2}\mathcal{N}\cos\left(\frac{\mu}{2}\right)^{\mathcal{N}-1}, \tag{15}$$

$$i\langle[S_z^{(m)}, U_{\mathrm{loc}}^\dagger S_y^{(m)}U_{\mathrm{loc}}]\rangle = -\frac{1}{2}\mathcal{N}\cos\left(\frac{\mu-\mu_{\mathrm{loc}}}{2}\right)^{\mathcal{N}-1}, \tag{16}$$

$$i\langle[S_z^{(m)}, U_{\mathrm{loc}}^\dagger S_z^{(m)}U_{\mathrm{loc}}]\rangle = 0. \tag{17}$$

The relative estimation uncertainty can be solve as ($\mu_{\mathrm{loc}}=\mu$ is chosen here, corresponding to optimal MAI evolution times when N is large.)

$$\begin{aligned}\xi_{\mathrm{MAI,loc}}^{-2}(\rho_{\mathrm{MS}}) = \frac{1}{4}\Big[&2+\cos\left(\frac{\mu}{2}\right)^{2\frac{N}{M}-4}\left(2+\left(-2+\frac{N}{M}\right)\frac{N}{M}-\left(-2+\frac{N}{M}\right)\frac{N}{M}\cos(\mu)\right)\\ &+\sqrt{4+\cos\left(\frac{\mu}{2}\right)^{4\frac{N}{M}-8}\left(2+\left(-2+\frac{N}{M}\right)\frac{N}{M}-\left(-2+\frac{N}{M}\right)\frac{N}{M}\cos(\mu)\right)^2+\cos\left(\frac{\mu}{2}\right)^{2\frac{N}{M}-4}\left(4\left(-2+\frac{N}{M}\right)\frac{N}{M}-4\left(2+\left(-2+\frac{N}{M}\right)\frac{N}{M}\right)\cos(\mu)\right)}\Big],\end{aligned} \tag{18}$$

which is determined by the local atom number in each node N/M.

Since there is no mode entanglement across nodes for ρ_{MS}, a diagonal squeezing matrix can be expressed as

$$\Xi^2(\rho_{\mathrm{MS}}) = \oplus_{m=1}^{M}\xi_m^2, \tag{19}$$

where ξ_m^{-2} is the maximum squeezing parameter on mode m. Thus, the covariance matrix is

$$\begin{aligned}\Sigma(\rho_{\mathrm{MS}}) &= \Sigma_{\mathrm{SN}}^{\frac{1}{2}}\Xi^2(\rho_{\mathrm{MS}})\Sigma_{\mathrm{SN}}^{\frac{1}{2}}\\ &= \oplus_{m=1}^{M}\frac{\xi_m^2}{N_m},\end{aligned} \tag{20}$$

where N_m is the local atom number on mode m. We assume the simple case where the optimal squeezing parameter ξ_m^2 and the total atom number $N_m=N/M$ are identical in each node, then we can finally obtain

$$\xi^{-2}(\rho_{\mathrm{MS}}) = \frac{\mathbf{n}^T\Sigma_{\mathrm{SN}}\mathbf{n}}{\mathbf{n}^T\Sigma(\rho_{\mathrm{MS}})\mathbf{n}} = \xi_m^{-2}(N/M). \tag{21}$$

B. Equal-weight combination estimation with nonlocal squeezing spin states ρ_{ME}

To generate the nonlocal squeezing spin states, we consider the nonlocal OAT interaction

$$\tilde{H}_{\mathrm{nl}} = \hbar\chi \left(\sum_{m=1}^{M} S_z^{(m)} \right)^2 . \tag{22}$$

This Hamiltonian can simultaneously generate mode entanglement and particle entanglement. The resulting mode entangled states are written as $\rho_{\mathrm{ME}} = |\psi_{\mathrm{ME}}\rangle\langle\psi_{\mathrm{ME}}|$, where $|\psi_{\mathrm{ME}}\rangle = e^{-i\frac{\tilde{H}_{\mathrm{nl}}}{\hbar}t}|\psi_0\rangle$.

1. Multiparameter moment matrix for ρ_{ME} in typical protocols using linear measurements

For mode entangled state ρ_{ME}, the covariance matrix is given as Eq. (16) in the main text, where the covariance matrix $\mathbf{\Gamma}_{mm}$ is

$$\mathbf{\Gamma}_{mm} = \begin{pmatrix} \mathrm{Cov}\left(S_y^{(m)}, S_y^{(m)}\right) & \mathrm{Cov}\left(S_y^{(m)}, S_z^{(m)}\right) \\ \mathrm{Cov}\left(S_z^{(m)}, S_y^{(m)}\right) & \mathrm{Cov}\left(S_z^{(m)}, S_z^{(m)}\right) \end{pmatrix}, \tag{23}$$

$$\mathrm{Cov}\left(S_y^{(m)}, S_y^{(m)}\right) = \frac{1}{8}\mathcal{N}\left(\mathcal{N} + 1 - (\mathcal{N}-1)\cos(\mu)^{M\mathcal{N}-2}\right), \tag{24}$$

$$\mathrm{Cov}\left(S_y^{(m)}, S_z^{(m)}\right) = \mathrm{Cov}\left(S_z^{(m)}, S_y^{(m)}\right) = \frac{1}{4}\mathcal{N}(\mathcal{N}-1)\cos\left(\frac{\mu}{2}\right)^{M\mathcal{N}-2}\sin\left(\frac{\mu}{2}\right), \tag{25}$$

$$\mathrm{Cov}\left(S_z^{(m)}, S_z^{(m)}\right) = \frac{\mathcal{N}}{4}, \tag{26}$$

and the covariance matrix $\mathbf{\Gamma}_{mn}$ is

$$\mathbf{\Gamma}_{mn} = \begin{pmatrix} \mathrm{Cov}\left(S_y^{(m)}, S_y^{(n)}\right) & \mathrm{Cov}\left(S_y^{(m)}, S_z^{(n)}\right) \\ \mathrm{Cov}\left(S_z^{(m)}, S_y^{(n)}\right) & \mathrm{Cov}\left(S_z^{(m)}, S_z^{(n)}\right) \end{pmatrix}, \tag{27}$$

$$\mathrm{Cov}\left(S_y^{(m)}, S_y^{(n)}\right) = \frac{1}{8}\mathcal{N}^2\left(1 - \cos(\mu)^{M\mathcal{N}-2}\right), \tag{28}$$

$$\mathrm{Cov}\left(S_y^{(m)}, S_z^{(n)}\right) = \mathrm{Cov}\left(S_z^{(m)}, S_y^{(n)}\right) = \frac{1}{4}\mathcal{N}^2\cos\left(\frac{\mu}{2}\right)^{M\mathcal{N}-2}\sin\left(\frac{\mu}{2}\right), \tag{29}$$

$$\mathrm{Cov}\left(S_z^{(m)}, S_z^{(n)}\right) = 0. \tag{30}$$

In the typical protocol, the commutator matrix can be expressed as $\boldsymbol{C} = \oplus_{m=1}^{M}\boldsymbol{C}_{mm}$, where $\boldsymbol{C}_{mm}$ is given as

$$\boldsymbol{C}_{mm} = i\begin{pmatrix} 0 & \langle[S_y^{(m)}, S_z^{(m)}]\rangle \\ \langle[S_z^{(m)}, S_y^{(m)}] & 0 \end{pmatrix}, \tag{31}$$

$$-i\langle[S_y^{(m)}, S_z^{(m)}]\rangle = i\langle[S_z^{(m)}, S_y^{(m)}]\rangle = \frac{1}{2}\mathcal{N}\cos\left(\frac{\mu}{2}\right)^{M\mathcal{N}-1} \tag{32}$$

The relative estimation uncertainty can be solve as

$$\xi_{\mathrm{L}}^{-2}(\rho_{\mathrm{ME}}) = \frac{4\cos(\mu/2)^{2N-2}\cos(\mu)^2}{-\left((-1+N)\cos(\mu)^N\right) + \cos(\mu)^2\left(3 + N - \sqrt{(-1+N)^2\left(-8\cos(\mu/2)^{2N-4}(-1+\cos(\mu)) + (\cos(\mu)^2 - \cos(\mu)^N)\sec(\mu)^4\right)}\right)}. \tag{33}$$

It is found that the sensitivity is independent with mode number M.

If a large total atom number $N \gg 1$ is considered, the maximum multiparameter sensitivity will be obtained as $\xi_{\mathrm{L}}^{-2}(\rho_{\mathrm{ME}}) \sim \frac{2}{3^{2/3}}N^{2/3}$ under the optimal evolution time $\mu_{\mathrm{L,opt}}(\rho_{\mathrm{ME}}) \sim 2\cdot 3^{1/6}N^{-2/3}$, which corresponds to the ones obtained from the single-parameter estimation scenarios [1, 2].

2. Multiparameter moment matrix for ρ_{ME} in nonlocal MAI protocols

The $2M \times 2M$ covariance matrix $\mathbf{\Gamma}$ can be expressed in terms of the submatrices $\mathbf{\Gamma}_{mm}, \mathbf{\Gamma}_{mn}$ as Eq. (16) in the main text, where

$$\mathbf{\Gamma}_{mm} = \begin{pmatrix} \mathrm{Cov}\left(U_{\mathrm{nl}}^{\dagger} S_y^{(m)} U_{\mathrm{nl}}, U_{\mathrm{nl}}^{\dagger} S_y^{(m)} U_{\mathrm{nl}}\right) & \mathrm{Cov}\left(U_{\mathrm{nl}}^{\dagger} S_y^{(m)} U_{\mathrm{nl}}, U_{\mathrm{nl}}^{\dagger} S_z^{(m)} U_{\mathrm{nl}}\right) \\ \mathrm{Cov}\left(U_{\mathrm{nl}}^{\dagger} S_z^{(m)} U_{\mathrm{nl}}, U_{\mathrm{nl}}^{\dagger} S_y^{(m)} U_{\mathrm{nl}}\right) & \mathrm{Cov}\left(U_{\mathrm{nl}}^{\dagger} S_z^{(m)} U_{\mathrm{nl}}, U_{\mathrm{nl}}^{\dagger} S_z^{(m)} U_{\mathrm{nl}}\right) \end{pmatrix}, \tag{34}$$

$$\mathrm{Cov}\left(U_{\mathrm{nl}}^{\dagger} S_y^{(m)} U_{\mathrm{nl}}, U_{\mathrm{nl}}^{\dagger} S_y^{(m)} U_{\mathrm{nl}}\right) = \frac{1}{8}\mathcal{N}\left(\mathcal{N} + 1 - (\mathcal{N} - 1)\cos(\mu - \mu_{\mathrm{nl}})^{M\mathcal{N}-2}\right), \tag{35}$$

$$\mathrm{Cov}\left(U_{\mathrm{nl}}^{\dagger} S_y^{(k)} U_{\mathrm{nl}}, U_{\mathrm{nl}}^{\dagger} S_z^{(m)} U_{\mathrm{nl}}\right) = \mathrm{Cov}\left(U_{\mathrm{nl}}^{\dagger} S_z^{(m)} U_{\mathrm{nl}}, U_{\mathrm{nl}}^{\dagger} S_y^{(m)} U_{\mathrm{nl}}\right) = \frac{1}{4}\mathcal{N}(\mathcal{N} - 1)\cos\left(\frac{\mu - \mu_{\mathrm{nl}}}{2}\right)^{M\mathcal{N}-2} \sin\left(\frac{\mu - \mu_{\mathrm{nl}}}{2}\right), \tag{36}$$

$$\mathrm{Cov}\left(U_{\mathrm{nl}}^{\dagger} S_z^{(m)} U_{\mathrm{nl}}, U_{\mathrm{nl}}^{\dagger} S_z^{(m)} U_{\mathrm{nl}}\right) = \frac{\mathcal{N}}{4}, \tag{37}$$

and

$$\Gamma_{mn} = \begin{pmatrix} \mathrm{Cov}\left(U_{\mathrm{nl}}^{\dagger} S_y^{(m)} U_{\mathrm{nl}}, U_{\mathrm{nl}}^{\dagger} S_y^{(n)} U_{\mathrm{nl}}\right) & \mathrm{Cov}\left(U_{\mathrm{nl}}^{\dagger} S_y^{(m)} U_{\mathrm{nl}}, U_{\mathrm{nl}}^{\dagger} S_z^{(n)} U_{\mathrm{nl}}\right) \\ \mathrm{Cov}\left(U_{\mathrm{nl}}^{\dagger} S_z^{(m)} U_{\mathrm{nl}}, U_{\mathrm{nl}}^{\dagger} S_y^{(n)} U_{\mathrm{nl}}\right) & \mathrm{Cov}\left(U_{\mathrm{nl}}^{\dagger} S_z^{(m)} U_{\mathrm{nl}}, U_{\mathrm{nl}}^{\dagger} S_z^{(n)} U_{\mathrm{nl}}\right) \end{pmatrix}, \tag{38}$$

$$\mathrm{Cov}\left(U_{\mathrm{nl}}^{\dagger} S_y^{(m)} U_{\mathrm{nl}}, U_{\mathrm{nl}}^{\dagger} S_y^{(n)} U_{\mathrm{nl}}\right) = \frac{1}{8}\mathcal{N}^2\left(1 - \cos(\mu - \mu_{\mathrm{nl}})^{M\mathcal{N}-2}\right), \tag{39}$$

$$\mathrm{Cov}\left(U_{\mathrm{nl}}^{\dagger} S_y^{(m)} U_{\mathrm{nl}}, U_{\mathrm{nl}}^{\dagger} S_z^{(n)} U_{\mathrm{nl}}\right) = \mathrm{Cov}\left(U_{\mathrm{nl}}^{\dagger} S_z^{(m)} U_{\mathrm{nl}}, U_{\mathrm{nl}}^{\dagger} S_y^{(n)} U_{\mathrm{nl}}\right) = \frac{1}{4}\mathcal{N}^2 \cos\left(\frac{\mu - \mu_{\mathrm{nl}}}{2}\right)^{M\mathcal{N}-2} \sin\left(\frac{\mu - \mu_{\mathrm{nl}}}{2}\right), \tag{40}$$

$$\mathrm{Cov}\left(U_{\mathrm{nl}}^{\dagger} S_z^{(m)} U_{\mathrm{nl}}, U_{\mathrm{nl}}^{\dagger} S_z^{(n)} U_{\mathrm{nl}}\right) = 0. \tag{41}$$

The commutator matrix $\boldsymbol{C}$ can be expressed by $\boldsymbol{C}_{mm}$ and $\boldsymbol{C}_{mn}$, where $\boldsymbol{C}_{mm}$ is a 2×2 submatrix with elements $(\boldsymbol{C}_{mm})_{ij} = i\langle[\mathcal{L}_i^{(m)}, \mathbf{X}_{\mathrm{MAI,nl},j}^{(m)}]\rangle$,

$$\boldsymbol{C}_{mm} = i\begin{pmatrix} \langle[S_y^{(m)}, U_{\mathrm{nl}}^{\dagger} S_y^{(m)} U_{\mathrm{nl}}]\rangle & \langle[S_y^{(m)}, U_{\mathrm{nl}}^{\dagger} S_z^{(m)} U_{\mathrm{nl}}]\rangle \\ \langle[S_z^{(m)}, U_{\mathrm{nl}}^{\dagger} S_y^{(m)} U_{\mathrm{nl}}]\rangle & \langle[S_z^{(m)}, U_{\mathrm{nl}}^{\dagger} S_z^{(m)} U_{\mathrm{nl}}]\rangle \end{pmatrix}, \tag{42}$$

$$i\langle[S_y^{(m)}, U_{\mathrm{nl}}^{\dagger} S_y^{(m)} U_{\mathrm{nl}}]\rangle = -\frac{1}{4}\mathcal{N}(\mathcal{N} - 1)\left(\cos\left(\mu - \frac{\mu_{\mathrm{nl}}}{2}\right)^{M\mathcal{N}-2} + \cos\left(\frac{\mu_{\mathrm{nl}}}{2}\right)^{M\mathcal{N}-2}\right)\sin\left(\frac{\mu_{\mathrm{nl}}}{2}\right), \tag{43}$$

$$i\langle[S_y^{(m)}, U_{\mathrm{nl}}^{\dagger} S_z^{(m)} U_{\mathrm{nl}}]\rangle = \frac{1}{2}\mathcal{N}\cos\left(\frac{\mu}{2}\right)^{M\mathcal{N}-1}, \tag{44}$$

$$i\langle[S_z^{(m)}, U_{\mathrm{nl}}^{\dagger} S_y^{(m)} U_{\mathrm{nl}}]\rangle = -\frac{1}{2}\mathcal{N}\cos\left(\frac{\mu - \mu_{\mathrm{nl}}}{2}\right)^{M\mathcal{N}-1}, \tag{45}$$

$$i\langle[S_z^{(m)}, U_{\mathrm{nl}}^{\dagger} S_z^{(m)} U_{\mathrm{nl}}]\rangle = 0. \tag{46}$$

The other submatrix $\boldsymbol{C}_{mn}$ whose elements are $(\boldsymbol{C}_{mn})_{ij} = i\langle[\mathcal{L}_i^{(m)}, \mathbf{X}_{\mathrm{MAI,nl},j}^{(n)}]\rangle$ is expressed as

$$\boldsymbol{C}_{mn} = i\begin{pmatrix} \langle[S_y^{(m)}, U_{\mathrm{nl}}^{\dagger} S_y^{(n)} U_{\mathrm{nl}}]\rangle & \langle[S_y^{(m)}, U_{\mathrm{nl}}^{\dagger} S_z^{(n)} U_{\mathrm{nl}}]\rangle \\ \langle[S_z^{(m)}, U_{\mathrm{nl}}^{\dagger} S_y^{(n)} U_{\mathrm{nl}}]\rangle & \langle[S_z^{(m)}, U_{\mathrm{nl}}^{\dagger} S_z^{(n)} U_{\mathrm{nl}}]\rangle \end{pmatrix}, \tag{47}$$

$$i\langle[S_y^{(m)}, U_{\mathrm{nl}}^{\dagger} S_y^{(n)} U_{\mathrm{nl}}]\rangle = -\frac{1}{4}\mathcal{N}^2\left(\cos\left(\mu - \frac{\mu_{\mathrm{nl}}}{2}\right)^{M\mathcal{N}-2} + \cos\left(\frac{\mu_{\mathrm{nl}}}{2}\right)^{M\mathcal{N}-2}\right)\sin\left(\frac{\mu_{\mathrm{nl}}}{2}\right), \tag{48}$$

$$\langle[S_y^{(m)}, U_{\mathrm{nl}}^{\dagger} S_z^{(n)} U_{\mathrm{nl}}]\rangle = i\langle[S_z^{(m)}, U_{\mathrm{nl}}^{\dagger} S_y^{(n)} U_{\mathrm{nl}}]\rangle = \langle[S_z^{(m)}, U_{\mathrm{nl}}^{\dagger} S_z^{(n)} U_{\mathrm{nl}}]\rangle = 0. \tag{49}$$

The relative estimation uncertainty can be solve as ($\mu_{\mathrm{nl}} = \mu$ is chosen here, corresponding to optimal MAI evolution times when N is large.)

$$\xi_{\mathrm{MAI,nl}}^{-2}(\rho_{\mathrm{ME}}) = \frac{1}{4}\Big[2 + \cos\left(\frac{\mu}{2}\right)^{2N-4}(2 + (-2+N)N - (-2+N)N\cos(\mu))$$
$$+ \sqrt{4 + \cos\left(\frac{\mu}{2}\right)^{4N-8}(2 + (-2+N)N - (-2+N)N\cos(\mu))^2 + \cos\left(\frac{\mu}{2}\right)^{2N-4}(4(-2+N)N - 4(2 + (-2+N)N)\cos(\mu))}\Big]. \tag{50}$$

It is found that the sensitivity is independent with the mode number M.

If a large total atom number $N \gg 1$ is considered, the optimal evolution time $\mu_{\mathrm{MAI(nl),opt}}(\rho_{\mathrm{ME}}) = 2N^{-1/2}$ will yield the Heisenberg-scaling sensitivity $\xi_{\mathrm{MAI,nl}}^{-2}(\rho_{\mathrm{ME}}) \sim N$, that will correspond to the single-parameter estimation scenarios [2].

3. Multiparameter moment matrix for ρ_{ME} in local MAI protocols

As in Eq. (16) in the main text, the $2M \times 2M$ covariance matrix $\mathbf{\Gamma}$ can be expressed by two submatrices $\mathbf{\Gamma}_{mm}, \mathbf{\Gamma}_{mn}$, which are expressed as

$$\mathbf{\Gamma}_{mm} = \begin{pmatrix} \mathrm{Cov}\left((U_{\mathrm{loc}}^{(m)})^\dagger S_y^{(m)} U_{\mathrm{loc}}^{(m)}, (U_{\mathrm{loc}}^{(m)})^\dagger S_y^{(m)} U_{\mathrm{loc}}^{(m)}\right) & \mathrm{Cov}\left((U_{\mathrm{loc}}^{(m)})^\dagger S_y^{(m)} U_{\mathrm{loc}}^{(m)}, (U_{\mathrm{loc}}^{(m)})^\dagger S_z^{(m)} U_{\mathrm{loc}}^{(m)}\right) \\ \mathrm{Cov}\left((U_{\mathrm{loc}}^{(m)})^\dagger S_z^{(m)} U_{\mathrm{loc}}^{(m)}, (U_{\mathrm{loc}}^{(m)})^\dagger S_y^{(m)} U_{\mathrm{loc}}^{(m)}\right) & \mathrm{Cov}\left((U_{\mathrm{loc}}^{(m)})^\dagger S_z^{(m)} U_{\mathrm{loc}}^{(m)}, (U_{\mathrm{loc}}^{(m)})^\dagger S_z^{(m)} U_{\mathrm{loc}}^{(m)}\right) \end{pmatrix}, \tag{51}$$

$$\mathrm{Cov}\left((U_{\mathrm{loc}}^{(m)})^\dagger S_y^{(m)} U_{\mathrm{loc}}^{(m)}, (U_{\mathrm{loc}}^{(m)})^\dagger S_y^{(m)} U_{\mathrm{loc}}^{(m)}\right) = \frac{1}{8}\mathcal{N}\left(1 + \mathcal{N} - (-1+\mathcal{N})\cos(\mu)^{(M-1)\mathcal{N}} \cos(\mu - \mu_{\mathrm{loc}})^{\mathcal{N}-2}\right), \tag{52}$$

$$\begin{aligned} &\mathrm{Cov}\left((U_{\mathrm{loc}}^{(m)})^\dagger S_y^{(m)} U_{\mathrm{loc}}^{(m)}, (U_{\mathrm{loc}}^{(m)})^\dagger S_z^{(m)} U_{\mathrm{loc}}^{(m)}\right) = \mathrm{Cov}\left((U_{\mathrm{loc}}^{(m)})^\dagger S_z^{(m)} U_{\mathrm{loc}}^{(m)}, (U_{\mathrm{loc}}^{(m)})^\dagger S_y^{(m)} U_{\mathrm{loc}}^{(m)}\right) \\ &= \frac{1}{4}\mathcal{N}(\mathcal{N}-1)\cos\left(\frac{\mu}{2}\right)^{(M-1)\mathcal{N}} \cos\left(\frac{\mu-\mu_{\mathrm{loc}}}{2}\right)^{\mathcal{N}-2} \sin\left(\frac{\mu-\mu_{\mathrm{loc}}}{2}\right), \end{aligned} \tag{53}$$

$$\mathrm{Cov}\left((U_{\mathrm{loc}}^{(m)})^\dagger S_z^{(m)} U_{\mathrm{loc}}^{(m)}, (U_{\mathrm{loc}}^{(m)})^\dagger S_z^{(m)} U_{\mathrm{loc}}^{(m)}\right) = \frac{\mathcal{N}}{4}, \tag{54}$$

and

$$\mathbf{\Gamma}_{mn} = \begin{pmatrix} \mathrm{Cov}\left((U_{\mathrm{loc}}^{(m)})^\dagger S_y^{(m)} U_{\mathrm{loc}}^{(m)}, (U_{\mathrm{loc}}^{(n)})^\dagger S_y^{(n)} U_{\mathrm{loc}}^{(n)}\right) & \mathrm{Cov}\left((U_{\mathrm{loc}}^{(m)})^\dagger S_y^{(m)} U_{\mathrm{loc}}^{(m)}, (U_{\mathrm{loc}}^{(n)})^\dagger S_z^{(n)} U_{\mathrm{loc}}^{(n)}\right) \\ \mathrm{Cov}\left((U_{\mathrm{loc}}^{(m)})^\dagger S_z^{(m)} U_{\mathrm{loc}}^{(m)}, (U_{\mathrm{loc}}^{(n)})^\dagger S_y^{(n)} U_{\mathrm{loc}}^{(n)}\right) & \mathrm{Cov}\left((U_{\mathrm{loc}}^{(m)})^\dagger S_z^{(m)} U_{\mathrm{loc}}^{(m)}, (U_{\mathrm{loc}}^{(n)})^\dagger S_z^{(n)} U_{\mathrm{loc}}^{(n)}\right) \end{pmatrix}, \tag{55}$$

$$\mathrm{Cov}\left((U_{\mathrm{loc}}^{(m)})^\dagger S_y^{(m)} U_{\mathrm{loc}}^{(m)}, (U_{\mathrm{loc}}^{(n)})^\dagger S_y^{(n)} U_{\mathrm{loc}}^{(n)}\right) = \frac{1}{8}\mathcal{N}^2\left(-\cos(\mu)^{(M-2)\mathcal{N}} \cos\left(\mu - \frac{\mu_{\mathrm{loc}}}{2}\right)^{2\mathcal{N}-2} + \cos\left(\frac{\mu_{\mathrm{loc}}}{2}\right)^{2\mathcal{N}-2}\right), \tag{56}$$

$$\begin{aligned} &\mathrm{Cov}\left((U_{\mathrm{loc}}^{(m)})^\dagger S_y^{(m)} U_{\mathrm{loc}}^{(m)}, (U_{\mathrm{loc}}^{(n)})^\dagger S_z^{(n)} U_{\mathrm{loc}}^{(n)}\right) = \mathrm{Cov}\left((U_{\mathrm{loc}}^{(m)})^\dagger S_z^{(m)} U_{\mathrm{loc}}^{(m)}, (U_{\mathrm{loc}}^{(n)})^\dagger S_y^{(n)} U_{\mathrm{loc}}^{(n)}\right) \\ &= \frac{1}{4}\mathcal{N}^2 \cos\left(\frac{\mu}{2}\right)^{(M-1)\mathcal{N}-1} \cos\left(\frac{\mu-\mu_{\mathrm{loc}}}{2}\right)^{\mathcal{N}-1} \sin\left(\frac{\mu}{2}\right), \end{aligned} \tag{57}$$

$$\mathrm{Cov}\left((U_{\mathrm{loc}}^{(m)})^\dagger S_z^{(m)} U_{\mathrm{loc}}^{(m)}, (U_{\mathrm{loc}}^{(n)})^\dagger S_z^{(n)} U_{\mathrm{loc}}^{(n)}\right) = 0. \tag{58}$$

The $2M \times 2M$ commutator matrix is $\boldsymbol{C} = \oplus_{m=1}^{M} \boldsymbol{C}_{mm}$, where $\boldsymbol{C}_{mm}$ is given as

$$\boldsymbol{C}_{mm} = i\begin{pmatrix} \langle [S_y^{(m)}, (U_{\mathrm{loc}}^{(m)})^\dagger S_y^{(m)} U_{\mathrm{loc}}^{(m)}]\rangle & \langle [S_y^{(m)}, (U_{\mathrm{loc}}^{(m)})^\dagger S_z^{(m)} U_{\mathrm{loc}}^{(m)}]\rangle \\ \langle [S_z^{(m)}, (U_{\mathrm{loc}}^{(m)})^\dagger S_y^{(m)} U_{\mathrm{loc}}^{(m)}]\rangle & \langle [S_y^{(m)}, (U_{\mathrm{loc}}^{(m)})^\dagger S_z^{(m)} U_{\mathrm{loc}}^{(m)}]\rangle \end{pmatrix}, \tag{59}$$

$$i\langle [S_y^{(m)}, (U_{\mathrm{loc}}^{(m)})^\dagger S_y^{(m)} U_{\mathrm{loc}}^{(m)}]\rangle = \frac{1}{4}\mathcal{N}(\mathcal{N}-1)\sin\left(\frac{\mu_{\mathrm{loc}}}{2}\right)\left(\cos(\mu)^{(M-1)\mathcal{N}} \cos\left(\mu - \frac{\mu_{\mathrm{loc}}}{2}\right)^{\mathcal{N}-2} + \cos\left(\frac{\mu_{\mathrm{loc}}}{2}\right)^{\mathcal{N}-2}\right), \tag{60}$$

$$i\langle [S_y^{(m)}, (U_{\mathrm{loc}}^{(m)})^\dagger S_z^{(k)} U_{loc}^{(k)}]\rangle = -\frac{1}{2}\mathcal{N}\cos\left(\frac{\mu}{2}\right)^{M\mathcal{N}-1}, \tag{61}$$

$$i\langle [S_z^{(m)}, (U_{\mathrm{loc}}^{(m)})^\dagger S_y^{(m)} U_{\mathrm{loc}}^{(m)}]\rangle = \frac{1}{2}\mathcal{N}\cos\left(\frac{\mu}{2}\right)^{(M-1)\mathcal{N}} \cos\left(\frac{\mu-\mu_{\mathrm{loc}}}{2}\right)^{\mathcal{N}-1}, \tag{62}$$

$$i\langle [S_z^{(m)}, (U_{\mathrm{loc}}^{(m)})^\dagger S_z^{(m)} U_{\mathrm{loc}}^{(m)}]\rangle = 0. \tag{63}$$

In Supplementary Figure 1, we compare the multiparameter squeezing detected from mode-separable states ρ_{MS} and mode-entangled states ρ_{ME}. For the spin states generated from the OAT interaction, the optimal metrological gain detected by a given readout strategy increases with the number of entangled particles N, while the optimal evolution time decreases with N. In our case, ρ_{ME} effectively behaves as an OAT state of $N = 100$ entangled particles, since the nonlocal interaction acts on the entire ensemble. By contrast, ρ_{MS} corresponds to a product of two independent OAT states with $N_A = N_B = 50$, whose metrological performance is yielded from a combination of two probe states with half atom number. Supplementary Figure 1 shows that when each probe is compared at its own optimal evolution times μ under the same measurement setting, ρ_{ME} yields a larger metrological gain than ρ_{MS}. In addition, due to the OAT property, ρ_{ME} reaches its maximum at a shorter time μ_{opt}.

C. Estimate arbitrary linear combinations of parameters with ρ_{ME}

In the previous sections Sec. I. A and B, we have focused on the estimation of the linear combination with equal magnitudes, i.e. estimation of $\Theta = \sum_{m=1}^{M} n_m \theta_m$ with $n_m = \pm\frac{1}{\sqrt{M}}$. However, a more flexible sensing strategies involving arbitrary linear combinations $\boldsymbol{n}$ is required in realistic multiparameter estimation tasks. In this section, we take $M = 2$ mode entangled states ρ_{ME} as an example, to show the metrological performance of MAI techniques in the general estimation scenario.

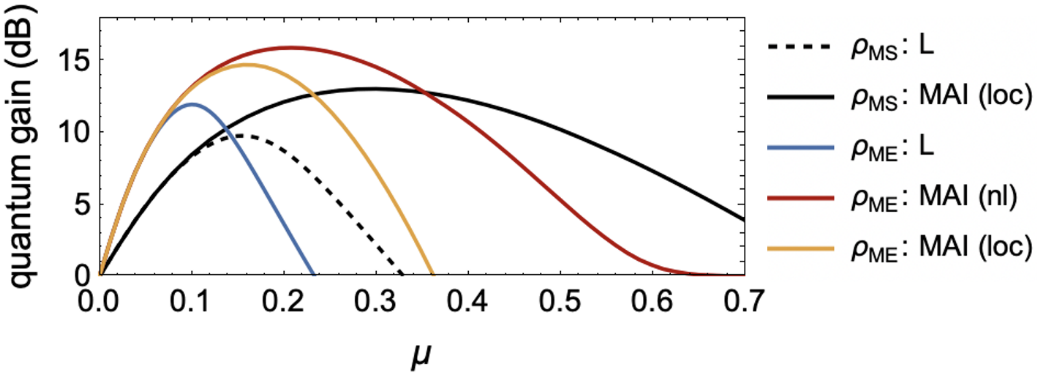

Supplementary Figure 1. Comparison of multiparameter squeezing for mode-separable states ρ_{MS} and mode-entangled states ρ_{ME} when the total atom number $N = 100$ and the mode number $M = 2$. It is observed that under the same measurement strategies, ρ_{MS} can achieve a higher sensitivity. It indicates that the entanglement between the modes leads to higher metrological sensitivity.

To estimate an arbitrary linear combination of two phases,

$$\Theta = \cos(\beta)\theta_A + \sin(\beta)\theta_B, \tag{64}$$

we find that the optimal particle distribution on two modes follows

$$\beta \sim \arctan(N_B/N_A), \tag{65}$$

where N_A, N_B are local particles on each modes. Here, the total particles are fixed $N = N_A + N_B$. Eq. (65) indicates that more particle resources are distributed to the larger-weighted mode. In the following, we provide the analytical expressions of covariance and commutator matrices and metrological gains, under linear measurements, nonlocal and local MAI strategies, respectively.

1. *linear measurements*

The covariance matrix is

$$\mathbf{\Gamma}_{\mathrm{L}}^{AB} = \begin{pmatrix} \mathrm{Cov}\left(S_y^A, S_y^A\right) & \mathrm{Cov}\left(S_y^A, S_z^A\right) & \mathrm{Cov}\left(S_y^A, S_y^B\right) & \mathrm{Cov}\left(S_y^A, S_z^B\right) \\ \mathrm{Cov}\left(S_z^A, S_y^A\right) & \mathrm{Cov}\left(S_z^A, S_z^A\right) & \mathrm{Cov}\left(S_z^A, S_y^B\right) & \mathrm{Cov}\left(S_z^A, S_z^B\right) \\ \mathrm{Cov}\left(S_y^B, S_y^A\right) & \mathrm{Cov}\left(S_y^B, S_z^A\right) & \mathrm{Cov}\left(S_y^B, S_y^B\right) & \mathrm{Cov}\left(S_y^B, S_z^B\right) \\ \mathrm{Cov}\left(S_z^B, S_y^A\right) & \mathrm{Cov}\left(S_z^B, S_z^A\right) & \mathrm{Cov}\left(S_z^B, S_y^B\right) & \mathrm{Cov}\left(S_z^B, S_z^B\right) \end{pmatrix} \tag{66}$$

with elements

$$\mathrm{Cov}\left(S_y^A, S_y^A\right) = \frac{1}{8}N_A\left(N_A + 1 - (N_A - 1)\cos(\mu)^{N-2}\right), \tag{67}$$

$$\mathrm{Cov}\left(S_y^A, S_z^A\right) = \frac{1}{4}N_A(N_A - 1)\cos(\mu/2)^{N-2}\sin(\mu/2), \tag{68}$$

$$\mathrm{Cov}\left(S_y^A, S_y^B\right) = \frac{1}{8}N_A(N - N_A)\left(-\cos(\mu)^{N-2} + 1\right), \tag{69}$$

$$\mathrm{Cov}\left(S_y^A, S_z^B\right) = \frac{1}{4}N_A(N - N_A)\cos(\mu/2)^{N-2}\sin(\mu/2), \tag{70}$$

$$\mathrm{Cov}\left(S_z^A, S_z^A\right) = \frac{N_A}{4}, \tag{71}$$

$$\mathrm{Cov}\left(S_z^A, S_y^B\right) = \frac{1}{4}N_A(N-N_A)\cos(\mu/2)^{N-2}\sin(\mu/2), \tag{72}$$

$$\mathrm{Cov}\left(S_z^A, S_z^B\right) = 0, \tag{73}$$

$$\mathrm{Cov}\left(S_y^B, S_y^B\right) = \frac{1}{8}(N_A-N)\left(-1+N_A-N-(1+N_A-N)\cos(\mu)^{N-2}\right), \tag{74}$$

$$\mathrm{Cov}\left(S_y^B, S_z^B\right) = \frac{1}{4}(N_A-N)(1+N_A-N)\cos(\mu/2)^{N-2}\sin(\mu/2), \tag{75}$$

$$\mathrm{Cov}\left(S_z^B, S_z^B\right) = \frac{1}{4}(N-N_A). \tag{76}$$

The remaining nonzero elements can be derived from the symmetry property of the covariance matrix, $\mathbf{\Gamma}_{\mathrm{L}}^{AB} = \left(\mathbf{\Gamma}_{\mathrm{L}}^{AB}\right)^T$.

The commutator matrix is

$$\boldsymbol{C}_{\mathrm{L}}^{AB} = \begin{pmatrix} 0 & i\langle[S_y^A, S_z^A]\rangle & 0 & 0 \\ i\langle[S_z^A, S_y^A]\rangle & 0 & 0 & 0 \\ 0 & 0 & 0 & i\langle[S_y^B, S_z^B]\rangle \\ 0 & 0 & i\langle[S_z^B, S_y^B]\rangle & 0 \end{pmatrix}, \tag{77}$$

with nonzero elements

$$-i\langle[\hat{S}_y^A, \hat{S}_z^A]\rangle = i\langle[\hat{S}_z^A, \hat{S}_y^A]\rangle = \frac{1}{2}N_A\cos(\mu/2)^{N-1}, \tag{78}$$

$$-i\langle\left[\hat{S}_y^B, \hat{S}_z^B\right]\rangle = i\langle\left[\hat{S}_z^B, \hat{S}_y^B\right]\rangle = \frac{1}{2}(N-N_A)\cos(\mu/2)^{N-1}, \tag{79}$$

$$\tag{80}$$

2. nonlocal MAI protocols

The covariance matrix is

$$\mathbf{\Gamma}_{\mathrm{MAL,nl}}^{AB} = \begin{pmatrix} \mathrm{Cov}\left(U_{\mathrm{nl}}^\dagger S_y^A U_{\mathrm{nl}}, U_{\mathrm{nl}}^\dagger S_y^A U_{\mathrm{nl}}\right) & \mathrm{Cov}\left(U_{\mathrm{nl}}^\dagger S_y^A U_{\mathrm{nl}}, U_{\mathrm{nl}}^\dagger S_z^A U_{\mathrm{nl}}\right) & \mathrm{Cov}\left(U_{\mathrm{nl}}^\dagger S_y^A U_{\mathrm{nl}}, U_{\mathrm{nl}}^\dagger S_y^B U_{\mathrm{nl}}\right) & \mathrm{Cov}\left(U_{\mathrm{nl}}^\dagger S_y^A U_{\mathrm{nl}}, U_{\mathrm{nl}}^\dagger S_z^B U_{\mathrm{nl}}\right) \\ \mathrm{Cov}\left(U_{\mathrm{nl}}^\dagger S_z^A U_{\mathrm{nl}}, U_{\mathrm{nl}}^\dagger S_y^A U_{\mathrm{nl}}\right) & \mathrm{Cov}\left(U_{\mathrm{nl}}^\dagger S_z^A U_{\mathrm{nl}}, U_{\mathrm{nl}}^\dagger S_z^A U_{\mathrm{nl}}\right) & \mathrm{Cov}\left(U_{\mathrm{nl}}^\dagger S_z^A U_{\mathrm{nl}}, U_{\mathrm{nl}}^\dagger S_y^B U_{\mathrm{nl}}\right) & \mathrm{Cov}\left(U_{\mathrm{nl}}^\dagger S_z^A U_{\mathrm{nl}}, U_{\mathrm{nl}}^\dagger S_z^B U_{\mathrm{nl}}\right) \\ \mathrm{Cov}\left(U_{\mathrm{nl}}^\dagger S_y^B U_{\mathrm{nl}}, U_{\mathrm{nl}}^\dagger S_y^A U_{\mathrm{nl}}\right) & \mathrm{Cov}\left(U_{\mathrm{nl}}^\dagger S_y^B U_{\mathrm{nl}}, U_{\mathrm{nl}}^\dagger S_z^A U_{\mathrm{nl}}\right) & \mathrm{Cov}\left(U_{\mathrm{nl}}^\dagger S_y^B U_{\mathrm{nl}}, U_{\mathrm{nl}}^\dagger S_y^B U_{\mathrm{nl}}\right) & \mathrm{Cov}\left(U_{\mathrm{nl}}^\dagger S_y^B U_{\mathrm{nl}}, U_{\mathrm{nl}}^\dagger S_z^B U_{\mathrm{nl}}\right) \\ \mathrm{Cov}\left(U_{\mathrm{nl}}^\dagger S_z^B U_{\mathrm{nl}}, U_{\mathrm{nl}}^\dagger S_y^A U_{\mathrm{nl}}\right) & \mathrm{Cov}\left(U_{\mathrm{nl}}^\dagger S_z^B U_{\mathrm{nl}}, U_{\mathrm{nl}}^\dagger S_z^A U_{\mathrm{nl}}\right) & \mathrm{Cov}\left(U_{\mathrm{nl}}^\dagger S_z^B U_{\mathrm{nl}}, U_{\mathrm{nl}}^\dagger S_y^B U_{\mathrm{nl}}\right) & \mathrm{Cov}\left(U_{\mathrm{nl}}^\dagger S_z^B U_{\mathrm{nl}}, U_{\mathrm{nl}}^\dagger S_z^B U_{\mathrm{nl}}\right) \end{pmatrix} \tag{81}$$

with elements

$$\mathrm{Cov}\left(U_{\mathrm{nl}}^\dagger S_y^A U_{\mathrm{nl}}, U_{\mathrm{nl}}^\dagger S_y^A U_{\mathrm{nl}}\right) = \frac{1}{8}N_A\left(N_A+1-(N_A-1)\cos(\mu-\mu_{\mathrm{nl}})^{N-2}\right), \tag{82}$$

$$\mathrm{Cov}\left(U_{\mathrm{nl}}^\dagger S_y^A U_{\mathrm{nl}}, U_{\mathrm{nl}}^\dagger S_z^A U_{\mathrm{nl}}\right) = \frac{1}{4}N_A(N_A-1)\cos((\mu-\mu_{\mathrm{nl}})/2)^{N-2}\sin((\mu-\mu_{\mathrm{nl}})/2), \tag{83}$$

$$\mathrm{Cov}\left(U_{\mathrm{nl}}^\dagger S_y^A U_{\mathrm{nl}}, U_{\mathrm{nl}}^\dagger S_y^B U_{\mathrm{nl}}\right) = \frac{1}{8}N_A(N-N_A)\left(-\cos(\mu-\mu_{\mathrm{nl}})^{N-2}+1\right), \tag{84}$$

$$\mathrm{Cov}\left(U_{\mathrm{nl}}^\dagger S_y^A U_{\mathrm{nl}}, U_{\mathrm{nl}}^\dagger S_z^B U_{\mathrm{nl}}\right) = \frac{1}{4}N_A(N-N_A)\cos((\mu-\mu_{\mathrm{nl}})/2)^{N-2}\sin((\mu-\mu_{\mathrm{nl}})/2), \tag{85}$$

$$\mathrm{Cov}\left(U_{\mathrm{nl}}^\dagger S_z^A U_{\mathrm{nl}}, U_{\mathrm{nl}}^\dagger S_z^A U_{\mathrm{nl}}\right) = \frac{N_A}{4}, \tag{86}$$

$$\mathrm{Cov}\left(U_{\mathrm{nl}}^\dagger S_z^A U_{\mathrm{nl}}, U_{\mathrm{nl}}^\dagger S_y^B U_{\mathrm{nl}}\right) = \frac{1}{4}N_A(N-N_A)\cos((\mu-\mu_{\mathrm{nl}})/2)^{N-2}\sin((\mu-\mu_{\mathrm{nl}})/2), \tag{87}$$

$$\mathrm{Cov}\left(U_{\mathrm{nl}}^\dagger S_z^A U_{\mathrm{nl}}, U_{\mathrm{nl}}^\dagger S_z^B U_{\mathrm{nl}}\right) = 0, \tag{88}$$

$$\mathrm{Cov}\left(U_{\mathrm{nl}}^\dagger S_y^B U_{\mathrm{nl}}, U_{\mathrm{nl}}^\dagger S_y^B U_{\mathrm{nl}}\right) = \frac{1}{8}(N_A-N)\left(-1+N_A-N-(1+N_A-N)\cos(\mu-\mu_{\mathrm{nl}})^{N-2}\right), \tag{89}$$

$$\mathrm{Cov}\left(U_{\mathrm{nl}}^\dagger S_y^B U_{\mathrm{nl}}, U_{\mathrm{nl}}^\dagger S_z^B U_{\mathrm{nl}}\right) = \frac{1}{4}(N_A-N)(1+N_A-N)\cos((\mu-\mu_{\mathrm{nl}})/2)^{N-2}\sin((\mu-\mu_{\mathrm{nl}})/2), \tag{90}$$

$$\mathrm{Cov}\left(U_{\mathrm{nl}}^\dagger S_z^B U_{\mathrm{nl}}, U_{\mathrm{nl}}^\dagger S_z^B U_{\mathrm{nl}}\right) = \frac{1}{4}(N-N_A). \tag{91}$$

The remaining nonzero elements can be derived from the symmetry property of the covariance matrix, $\mathbf{\Gamma}^{AB}_{\mathrm{MAI,nl}} = \left(\mathbf{\Gamma}^{AB}_{\mathrm{MAI,nl}}\right)^T$.

The commutator matrix is

$$\boldsymbol{C}^{AB}_{\mathrm{MAI,nl}} = i\begin{pmatrix} \langle[S^A_y, U^\dagger_{\mathrm{nl}}S^A_yU_{\mathrm{nl}}]\rangle & \langle[S^A_y, U^\dagger_{\mathrm{nl}}S^A_zU_{\mathrm{nl}}]\rangle & \langle[S^A_y, U^\dagger_{\mathrm{nl}}S^B_yU_{\mathrm{nl}}] & \langle[S^A_y, U^\dagger_{\mathrm{nl}}S^B_zU_{\mathrm{nl}}] \\ \langle[S^A_z, U^\dagger_{\mathrm{nl}}S^A_yU_{\mathrm{nl}}]\rangle & \langle[S^A_z, U^\dagger_{\mathrm{nl}}S^A_zU_{\mathrm{nl}}]\rangle & \langle[S^A_z, U^\dagger_{\mathrm{nl}}S^B_yU_{\mathrm{nl}}] & \langle[S^A_z, U^\dagger_{\mathrm{nl}}S^B_zU_{\mathrm{nl}}] \\ \langle[S^B_y, U^\dagger_{\mathrm{nl}}S^A_yU_{\mathrm{nl}}]\rangle & \langle[S^B_y, U^\dagger_{\mathrm{nl}}S^A_zU_{\mathrm{nl}}]\rangle & \langle[S^B_y, U^\dagger_{\mathrm{nl}}S^B_yU_{\mathrm{nl}}] & \langle[S^B_y, U^\dagger_{\mathrm{nl}}S^B_zU_{\mathrm{nl}}] \\ \langle[S^B_z, U^\dagger_{\mathrm{nl}}S^A_yU_{\mathrm{nl}}]\rangle & \langle[S^B_z, U^\dagger_{\mathrm{nl}}S^A_zU_{\mathrm{nl}}]\rangle & \langle[S^B_z, U^\dagger_{\mathrm{nl}}S^B_yU_{\mathrm{nl}}] & \langle[S^B_z, U^\dagger_{\mathrm{nl}}S^B_zU_{\mathrm{nl}}] \end{pmatrix}, \tag{92}$$

with elements

$$i\langle[S^A_y, U^\dagger_{\mathrm{nl}}S^A_yU_{\mathrm{nl}}]\rangle = \frac{1}{4}(N_A-1)N_A\left(\cos^{N-2}\left(\mu-\frac{\mu_{\mathrm{nl}}}{2}\right)+\cos^{N-2}\left(\frac{\mu_{\mathrm{nl}}}{2}\right)\right)\sin\left(\frac{\mu_{\mathrm{nl}}}{2}\right), \tag{93}$$

$$i\langle[S^A_y, U^\dagger_{\mathrm{nl}}S^A_zU_{\mathrm{nl}}]\rangle = -\frac{1}{2}N_A\cos^{N-1}\left(\frac{\mu}{2}\right), \tag{94}$$

$$i\langle[S^A_y, U^\dagger_{\mathrm{nl}}S^B_yU_{\mathrm{nl}}]\rangle = \frac{1}{4}N_A(N-N_A)\left[\cos^{N-2}\left(\mu-\frac{\mu_{\mathrm{nl}}}{2}\right)+\cos^{N-2}\left(\frac{\mu_{\mathrm{nl}}}{2}\right)\right]\sin\left(\frac{\mu_{\mathrm{nl}}}{2}\right), \tag{95}$$

$$i\langle[S^A_y, U^\dagger_{\mathrm{nl}}S^B_zU_{\mathrm{nl}}]\rangle = 0, \tag{96}$$

$$i\langle[S^A_z, U^\dagger_{\mathrm{nl}}S^A_yU_{\mathrm{nl}}]\rangle = \frac{1}{2}N_A\cos^{N-1}\left(\frac{1}{2}(\mu-\mu_{\mathrm{nl}})\right), \tag{97}$$

$$i\langle[S^A_z, U^\dagger_{\mathrm{nl}}S^A_zU_{\mathrm{nl}}]\rangle = 0, \tag{98}$$

$$i\langle[S^A_z, U^\dagger_{\mathrm{nl}}S^B_yU_{\mathrm{nl}}]\rangle = 0, \tag{99}$$

$$i\langle[S^A_z, U^\dagger_{\mathrm{nl}}S^B_zU_{\mathrm{nl}}]\rangle = 0, \tag{100}$$

$$i\langle[S^B_y, U^\dagger_{\mathrm{nl}}S^A_yU_{\mathrm{nl}}]\rangle = \frac{1}{4}N_A(N-N_A)\left[\cos^{N-2}\left(\mu-\frac{\mu_{\mathrm{nl}}}{2}\right)+\cos^{N-2}\left(\frac{\mu_{\mathrm{nl}}}{2}\right)\right]\sin\left(\frac{\mu_{\mathrm{nl}}}{2}\right), \tag{101}$$

$$i\langle[S^B_y, U^\dagger_{\mathrm{nl}}S^A_zU_{\mathrm{nl}}]\rangle = 0, \tag{102}$$

$$i\langle[S^B_y, U^\dagger_{\mathrm{nl}}S^B_yU_{\mathrm{nl}}]\rangle = \frac{1}{4}(N_A-N)(1+N_A-N)\left[\cos^{N-2}\left(\mu-\frac{\mu_{\mathrm{nl}}}{2}\right)+\cos^{N-2}\left(\frac{\mu_{\mathrm{nl}}}{2}\right)\right]\sin\left(\frac{\mu_{\mathrm{nl}}}{2}\right), \tag{103}$$

$$i\langle[S^B_y, U^\dagger_{\mathrm{nl}}S^B_zU_{\mathrm{nl}}]\rangle = \frac{1}{2}(N_A-N)\cos^{N-1}\left(\frac{\mu}{2}\right), \tag{104}$$

$$i\langle[S^B_z, U^\dagger_{\mathrm{nl}}S^A_yU_{\mathrm{nl}}]\rangle = 0, \tag{105}$$

$$i\langle[S^B_z, U^\dagger_{\mathrm{nl}}S^A_zU_{\mathrm{nl}}]\rangle = 0, \tag{106}$$

$$i\langle[S^B_z, U^\dagger_{\mathrm{nl}}S^B_yU_{\mathrm{nl}}]\rangle = \frac{1}{2}(N-N_A)\cos^{N-1}\left(\frac{1}{2}(\mu-\mu_{\mathrm{nl}})\right), \tag{107}$$

$$i\langle[S^B_z, U^\dagger_{\mathrm{nl}}S^B_zU_{\mathrm{nl}}]\rangle = 0. \tag{108}$$

3. local MAI protocols

The local MAI unitary can be expressed as $U_{\mathrm{loc}} = U_A \otimes U_B$ with $U_A = e^{-i(S^A_z)^2\mu^A_{\mathrm{loc}}/2}$ and $U_B = e^{-i(S^B_z)^2\mu^B_{\mathrm{loc}}/2}$. To investigate a more general case with arbitrary distribution N_A, N_B, the local evolution times $\mu^A_{\mathrm{loc}}, \mu^B_{\mathrm{loc}}$ need to be optimized individually.

The covariance matrix is

$$\mathbf{\Gamma}^{AB}_{\mathrm{MAI,loc}} = \begin{pmatrix} \mathrm{Cov}\left(U^\dagger_AS^A_yU_A, U^\dagger_AS^A_yU_A\right) & \mathrm{Cov}\left(U^\dagger_AS^A_yU_A, U^\dagger_AS^A_zU_A\right) & \mathrm{Cov}\left(U^\dagger_AS^A_yU_A, U^\dagger_BS^B_yU_B\right) & \mathrm{Cov}\left(U^\dagger_AS^A_yU_A, U^\dagger_BS^B_zU_B\right) \\ \mathrm{Cov}\left(U^\dagger_AS^A_zU_A, U^\dagger_AS^A_yU_A\right) & \mathrm{Cov}\left(U^\dagger_AS^A_zU_A, U^\dagger_AS^A_zU_A\right) & \mathrm{Cov}\left(U^\dagger_AS^A_zU_A, U^\dagger_BS^B_yU_B\right) & \mathrm{Cov}\left(U^\dagger_AS^A_zU_A, U^\dagger_BS^B_zU_B\right) \\ \mathrm{Cov}\left(U^\dagger_BS^B_yU_B, U^\dagger_AS^A_yU_A\right) & \mathrm{Cov}\left(U^\dagger_BS^B_yU_B, U^\dagger_AS^A_zU_A\right) & \mathrm{Cov}\left(U^\dagger_BS^B_yU_B, U^\dagger_BS^B_yU_B\right) & \mathrm{Cov}\left(U^\dagger_BS^B_yU_B, U^\dagger_BS^B_zU_B\right) \\ \mathrm{Cov}\left(U^\dagger_BS^B_zU_B, U^\dagger_AS^A_yU_A\right) & \mathrm{Cov}\left(U^\dagger_BS^B_zU_B, U^\dagger_AS^A_zU_A\right) & \mathrm{Cov}\left(U^\dagger_BS^B_zU_B, U^\dagger_BS^B_yU_B\right) & \mathrm{Cov}\left(U^\dagger_BS^B_zU_B, U^\dagger_BS^B_zU_B\right) \end{pmatrix} \tag{109}$$

with elements

$$
\begin{aligned}
&\mathrm{Cov}\left(U_A^\dagger S_y^A U_A, U_A^\dagger S_y^A U_A\right) = \frac{1}{8}N_A\left[1+N_A-(-1+N_A)\cos^{N-N_A}(\mu)\cos^{N_A-2}(\mu-\mu_{\mathrm{loc}}^A)\right],\\
&\mathrm{Cov}\left(U_A^\dagger S_y^A U_A, U_A^\dagger S_z^A U_A\right) = \frac{1}{4}N_A(N_A-1)\cos^{N-N_A}\left(\frac{\mu}{2}\right)\cos^{N_A-1}\left(\frac{1}{2}(\mu-\mu_{\mathrm{loc}}^A)\right)\tan\left(\frac{1}{2}(\mu-\mu_{\mathrm{loc}}^A)\right),\\
&\mathrm{Cov}\left(U_A^\dagger S_y^A U_A, U_B^\dagger S_y^B U_B\right) = \frac{1}{8}N_A(2N-N_A)\left(-\cos\left(\mu-\frac{\mu_{\mathrm{loc}}^A}{2}\right)^{N_A-1}\cos\left(\mu-\frac{\mu_{\mathrm{loc}}^B}{2}\right)^{N-N_A-1}+\cos\left(\frac{\mu_{\mathrm{loc}}^A}{2}\right)^{N_A-1}\cos\left(\frac{\mu_{\mathrm{loc}}^B}{2}\right)^{N-N_A-1}\right),\\
&\mathrm{Cov}\left(U_A^\dagger S_y^A U_A, U_B^\dagger S_z^B U_B\right) = \frac{1}{4}N_A(N-N_A)\cos^{N-N_A-1}\left(\frac{\mu}{2}\right)\cos^{N_A-1}\left(\frac{\mu-\mu_{\mathrm{loc}}^A}{2}\right)\sin\left(\frac{\mu}{2}\right),\\
&\mathrm{Cov}\left(U_A^\dagger S_z^A U_A, U_A^\dagger S_z^A U_A\right) = \frac{N_A}{4},\\
&\mathrm{Cov}\left(U_A^\dagger S_z^A U_A, U_B^\dagger S_y^B U_B\right) = \frac{1}{4}N_A(N-N_A)\cos^{N_A-1}\left(\frac{\mu}{2}\right)\cos^{N-N_A-1}\left(\frac{\mu-\mu_{\mathrm{loc}}^B}{2}\right)\sin\left(\frac{\mu}{2}\right),\\
&\mathrm{Cov}\left(U_A^\dagger S_z^A U_A, U_B^\dagger S_z^B U_B\right) = 0,\\
&\mathrm{Cov}\left(U_B^\dagger S_y^B U_B, U_B^\dagger S_y^B U_B\right) = \frac{1}{8}(N-N_A)\left[1+N-N_A-(-1-N+N_A))\cos^{N_A}(\mu)\cos^{N-N_A-2}(\mu-\mu_{\mathrm{loc}}^B)\right],\\
&\mathrm{Cov}\left(U_B^\dagger S_y^B U_B, U_B^\dagger S_z^B U_B\right) = \frac{1}{4}(N-N_A-1)(N-N_A)\cos^{N_A}\left(\frac{\mu}{2}\right)\cos^{N-N_A-1}\left(\frac{1}{2}(\mu-\mu_{\mathrm{loc}}^B)\right)\tan\left(\frac{1}{2}(\mu-\mu_{\mathrm{loc}}^B)\right),\\
&\mathrm{Cov}\left(U_B^\dagger S_z^B U_B, U_B^\dagger S_z^B U_B\right) = \frac{N-N_A}{4}.
\end{aligned}
\tag{110}
$$

The remaining elements can be derived from the symmetry property of the covariance matrix, $\mathbf{\Gamma}_{\mathrm{MAI,loc}}^{AB} = \left(\mathbf{\Gamma}_{\mathrm{MAI,loc}}^{AB}\right)^T$.

The commutator matrix is

$$
C_{\mathrm{MAI,loc}}^{AB} = i\begin{pmatrix}
\langle[S_y^A, U_A^\dagger S_y^A U_A]\rangle & \langle[S_y^A, U_A^\dagger S_z^A U_A]\rangle & 0 & 0\\
\langle[S_z^A, U_A^\dagger S_y^A U_A]\rangle & \langle[S_z^A, U_A^\dagger S_z^A U_A]\rangle & 0 & 0\\
0 & 0 & \langle[S_y^B, U_B^\dagger S_y^B U_B] & \langle[S_y^B, U_B^\dagger S_z^B U_B]\\
0 & 0 & \langle[S_z^B, U_B^\dagger S_y^B U_B] & \langle[S_z^B, U_B^\dagger S_z^B U_B]
\end{pmatrix},
\tag{111}
$$

with elements

$$
i\langle[S_y^A, U_A^\dagger S_y^A U_A]\rangle = \frac{1}{4}N_A(N_A-1)\sin\left(\frac{\mu_{\mathrm{loc}}^A}{2}\right)\left[\cos^{N_A-2}\left(\frac{\mu_{\mathrm{loc}}^A}{2}\right)+\cos^{N-N_A}(\mu)\cos^{N_A-2}\left(\mu-\frac{\mu_{\mathrm{loc}}^A}{2}\right)\right],
\tag{112}
$$

$$
i\langle[S_y^A, U_A^\dagger S_z^A U_A]\rangle = -\frac{1}{2}N_A\cos^{N-1}\left(\frac{\mu}{2}\right),
\tag{113}
$$

$$
i\langle[S_z^A, U_A^\dagger S_y^A U_A]\rangle = \frac{1}{2}N_A\cos^{N-N_A}\left(\frac{\mu}{2}\right)\cos^{N_A-1}\left(\frac{\mu-\mu_{\mathrm{loc}}^A}{2}\right),
\tag{114}
$$

$$
i\langle[S_z^A, U_A^\dagger S_z^A U_A]\rangle = 0,
\tag{115}
$$

$$
i\langle[S_y^B, U_B^\dagger S_y^B U_B]\rangle = \frac{1}{4}(N-N_A-1)(N-N_A)\sin\left(\frac{\mu_{\mathrm{loc}}^B}{2}\right)\left[\cos^{N-N_A-2}\left(\frac{\mu_{\mathrm{loc}}^B}{2}\right)+\cos^{N_A}(\mu)\cos^{N-N_A-2}\left(\mu-\frac{\mu_{\mathrm{loc}}^B}{2}\right)\right],
\tag{116}
$$

$$
i\langle[S_y^B, U_B^\dagger S_z^B U_B]\rangle = -\frac{1}{2}(N-N_A)\cos^{N-1}\left(\frac{\mu}{2}\right),
\tag{117}
$$

$$
i\langle[S_z^B, U_B^\dagger S_y^B U_B]\rangle = \frac{1}{2}(N-N_A)\cos^{N_A}\left(\frac{\mu}{2}\right)\cos^{N-N_A-1}\left(\frac{\mu-\mu_{\mathrm{loc}}^B}{2}\right),
\tag{118}
$$

$$
i\langle[S_z^B, U_B^\dagger S_z^B U_B]\rangle = 0.
\tag{119}
$$

Based on the analytical expressions in three strategies, we plot the maximum metrological gains to estimate $\Theta = \cos(\beta)\theta_A + \sin(\beta)\theta_B$ in Supplementary Figure 2. In Supplementary Figure 2(a), we observe that the maximal sensitivities detected by linear measurements and nonlocal MAI strategy are independent with different combination β, whereas the metrological gain from local MAI changes with β. This is because local MAI protocols involve independent local OAT evolutions on each mode. When β changes, the optimal distribution of local particles N_A, N_B must be adjusted accordingly. In this case, applying independent local

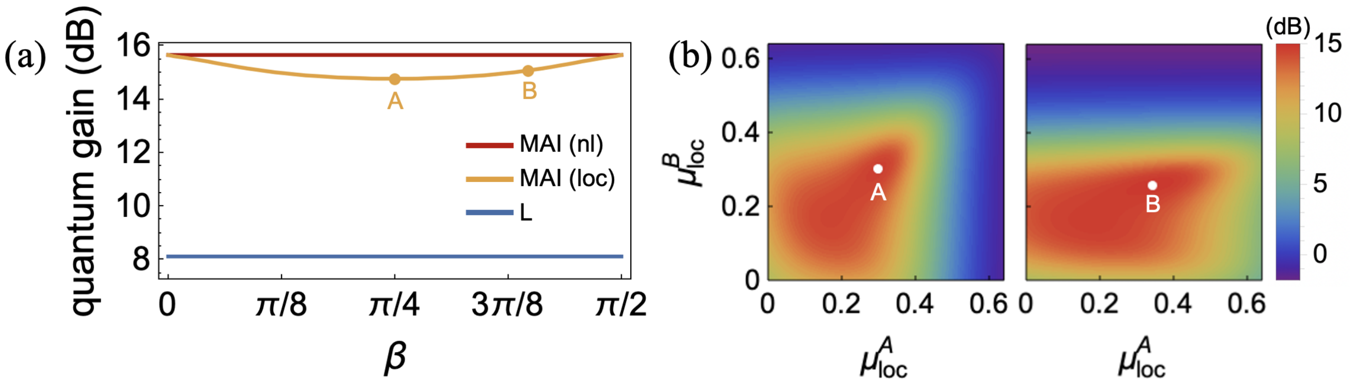

Supplementary Figure 2. Quantum gain of estimating a specific linear combination of phases $\Theta = \cos(\beta)\theta_A + \sin(\beta)\theta_B$, for $M = 2$ mode entangled states $\rho_{\rm ME}$. (a) Considering $\rho_{\rm ME}$ with total atom number $N = 100$ and state preparation times $\mu = 0.16$, we set the optimal distribution (N_A, N_B) and plot corresponding metrological gains from different detection strategies as a functions of combination variable β. (b) Local operations $U_A = e^{-i(S_z^A)^2\mu_2^A/2}$ and $U_B = e^{-i(S_z^B)^2\mu_2^B/2}$ are used in local MAI strategies. We show the sensitivity changes with MAI times μ_2^A, μ_2^B at (left) $\beta = \pi/4, N_A = N_B = 50$ and (right) $\beta = \arctan(3)$ and $N_A = 25, N_B = 75$, where the maximum sensitivies are labeled as "A" and "B".

evolutions on each mode leads to different entanglement properties. In Supplementary Figure 2(b), we show optimal evolution times $\mu^A_{\rm loc}, \mu^B_{\rm loc}$ under different combination β. In particular, for the symmetric case with $\beta = \pi/4$ and $N_A = N_B = 50$, the optimal times are the same $\mu^A_{\rm loc} = \mu^B_{\rm loc}$. In this case, local MAI yields the smallest gain, because the amount of mode entanglement left unexploited by local MAI operations is the largest. When the weight is biased, the optimal times $\mu^A_{\rm loc}, \mu^B_{\rm loc}$ are different. In an extreme case of $\beta = \pi/2$ and $N_A = 0, N_B = 100$, the entanglement resources effectively concentrate in a single mode. The local MAI operations effectively act on the full N entangled particles, yielding the maximum sensitivity which is the same as the one from the nonlocal MAI strategy.

SUPPLEMENTARY NOTE 2. QUANTITATIVE ANALYSIS OF NON-GAUSSIAN PROPERTIES IN OAT EVOLUTION

A key advantage of MAI strategies is their enhanced capability to reveal non-Gaussian sensitivity, for which standard linear measurements may fail to capture these higher-order moments. In this section, we provide a quantitative characterization of the transformation, from Gaussian to non-Gaussian, during the OAT evolution.

Although multi-mode spin states, $\rho_{\rm ME}$ and $\rho_{\rm MS}$, are involved in multiparameter estimation tasks, here we analyze the single-mode case for a more intuitive understanding, since it indicates the essential mechanism for generating non-Gaussianity in both multi-mode scenarios. Staring from a coherent spin state along the x-direction $|\psi_0\rangle$, the OAT interaction $H = \hbar\chi S_z^2$ yields the spin squeezed states $|\psi_{\rm OAT}\rangle = e^{-iS_z^2\mu/2}|\psi_0\rangle$, where $\mu = 2\chi t$ is an adimensional interaction time.

We first quantify non-Gaussianity by the excess kurtosis [3, 4]. This quantity is determined by up to fourth-order moment of a collective spin $S_{\boldsymbol{a}}$ along the direction $\boldsymbol{a}$, which is expressed in terms of

$$K_{\rm ex} = \min_{\boldsymbol{a}} \left(\frac{\langle (S_{\boldsymbol{a}} - \langle S_{\boldsymbol{a}} \rangle)^4 \rangle}{\langle (S_{\boldsymbol{a}} - \langle S_{\boldsymbol{a}} \rangle)^2 \rangle^2} - 3 \right). \tag{120}$$

A negative value $K_{\rm ex} < 0$ reveals non-Gaussianity in the collective-spin measurement statistics.

Non-Gaussianity can also be characterized through negativity of a quasi-probability distribution. Therefore we quantify the Wigner negativity, defined as the double volume of the negative part of the Wigner function. For a $j = N/2$ spin state, this quantity reads [5]

$$WN = \frac{1}{2} \left(\frac{2j+1}{4\pi} \int_{\theta=0}^{\pi} \int_{\phi=0}^{2\pi} |W_\rho(\theta, \phi)| \sin\theta d\theta d\phi - 1 \right), \tag{121}$$

where $W_\rho(\theta, \phi)$ denotes the Wigner function value at point (θ, ϕ) on the Bloch sphere. The presence of negative regions in Wigner distribution, i.e. $WN > 0$, reveals non-classicality of the states [6].

In Supplementary Figure 3, we demonstrate the state evolution for a single-mode squeezed state $|\psi_{\rm OAT}\rangle = e^{-iS_z^2\mu/2}$ and the metrological sensitivity of a relevant two-mode nonlocal squeezed state $|\psi_{\rm ME}\rangle = e^{-i(S_z^A+S_z^B)^2\mu/2}$, for fixed total atoms $N = 100$.

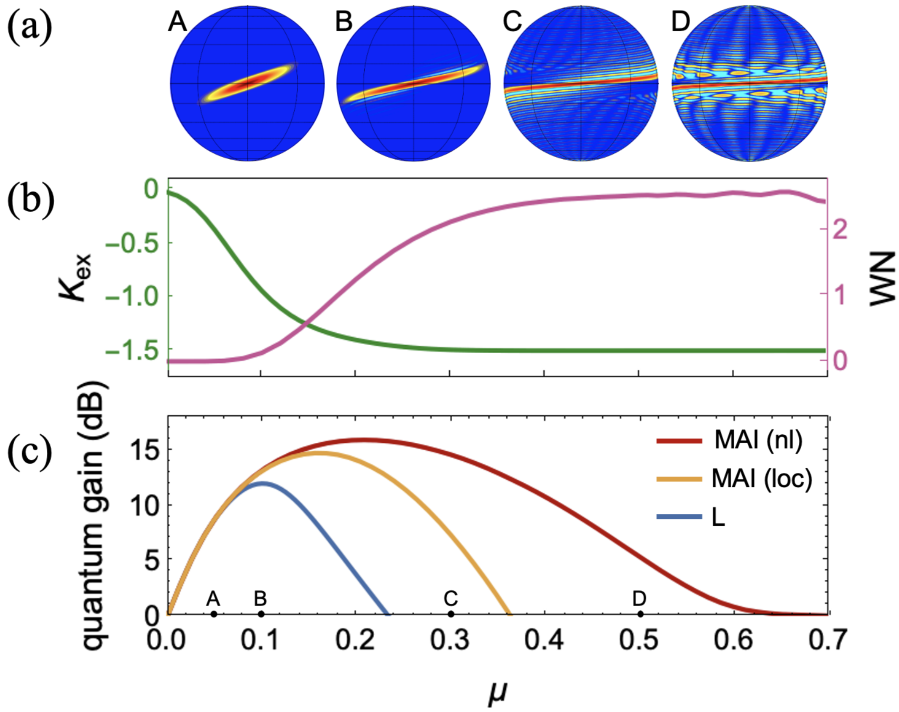

Supplementary Figure 3. Quantify non-Gaussianity and metrological sensitivity of spin OAT states with total atom number $N = 100$. (a) Wigner plots for the single-mode spin squeezed states $|\psi_{\mathrm{OAT}}\rangle$ at $\mu = \{0.05, 0.1, 0.3, 0.5\}$. As OAT squeezing μ increases, the states change from Gaussian spin squeezed states to over-squeezed non-Gaussian spin states. (b) Excess kurtosis ($K_{\mathrm{ex}} < 0$ indicates non-Gaussianity) and Wigner negativity ($WN > 0$ indicates non-classicality) are considered to quantify the non-Gaussianity of $|\psi_{\mathrm{OAT}}\rangle$. (c) If $|\psi_{\mathrm{OAT}}\rangle$ is evenly distributed into two modes, the metrological sensitivity of the resulting states $|\psi_{\mathrm{ME}}\rangle$ revealed from linear measurements and MAI protocols.

Supplementary Figure 3(a) shows Wigner functions of $|\psi_{\mathrm{OAT}}\rangle$ at different OAT times. It is observed that at small times μ, Wigner function is approximately elliptical shape, corresponding to Gaussian spin squeezed states. As μ increases, the distribution is twisted and develops negative regions and interference fringes, indicating that the states are over-squeezed and evolve in strongly non-Gaussian regimes. To quantify this transformation, we plot the excess kurtosis and Wigner negativity as a function of OAT times μ in Supplementary Figure 3(b). The emergence and growth of $K_{\mathrm{ex}} < 0$ and $WN > 0$ indicate that the state becomes more non-Gaussian and non-classical as μ increases. If this resource $|\psi_{\mathrm{OAT}}\rangle$ is spatially distributed into two modes, it becomes the mode entangled states $|\psi_{\mathrm{ME}}\rangle$ whose metrological sensitivities from different detection strategies are presented in Supplementary Figure 3(c). It is clearly shown that both MAI strategies are able to reach higher metrological gain in the large-μ non-Gaussian regime, where typical linear measurements rapidly become invalid.

SUPPLEMENTARY NOTE 3. MULTIPARAMETER ESTIMATION FOR CONTINUOUS-VARIABLE SYSTEMS

A. Detection noise effects on two-mode squeezed states

A two mode squeezed vacuum state can be prepared by a two mode squeezing operator $S_{\mathrm{AB}} = e^{-\zeta a^\dagger b^\dagger + \zeta^* ab}$, with the squeezing parameter $\zeta = re^{i\phi}$. Without loss of generality, we assume $\phi = 0$. A complete family of linear operator is made of $\mathcal{L} = (x, p)$, where $x = (a + a^\dagger)/\sqrt{2}$ and $p = -i(a - a^\dagger)/\sqrt{2}$ are quadrature observables. Therefore, the family of linear measurements over two-mode reads $\mathcal{A}_{\mathrm{L}} = (x_A, p_A, x_B, p_B)$. In the typical scenario where only linear measurements are considered, the covariance

and commutator matrix are

$$\mathbf{\Gamma} = \frac{1}{2}\begin{pmatrix} \cosh(2r) & 0 & -\sinh(2r) & 0 \\ 0 & \cosh(2r) & 0 & \sinh(2r) \\ -\sinh(2r) & 0 & \cosh(2r) & 0 \\ 0 & \sinh(2r) & 0 & \cosh(2r) \end{pmatrix}, \tag{122}$$

$$\boldsymbol{C} = \begin{pmatrix} 0 & 1 & 0 & 0 \\ -1 & 0 & 0 & 0 \\ 0 & 0 & 0 & 1 \\ 0 & 0 & -1 & 0 \end{pmatrix}. \tag{123}$$

After optimizing the measurements, we obtain the maximum sensitivity

$$\xi_{\mathrm{L}}^{-2} = e^{2r}. \tag{124}$$

Then, we investigate the MAI strategies, where an additional evolution U_{MAI} is introduced before linear measurements. We first consider a nonlocal evolution, which is performed by a two-mode squeezing operator $U_{\mathrm{nl}} = e^{r_{\mathrm{nl}}(a^\dagger b^\dagger - ab)}$. Based on the associated family $\mathcal{A}_{\mathrm{MAI}} = \left(U_{\mathrm{nl}}^\dagger x_A U_{\mathrm{nl}}, U_{\mathrm{nl}}^\dagger p_A U_{\mathrm{nl}}, U_{\mathrm{nl}}^\dagger x_B U_{\mathrm{nl}}, U_{\mathrm{nl}}^\dagger p_B U_{\mathrm{nl}}\right)$, the covariance and commutator matrices can be written as

$$\mathbf{\Gamma} = \frac{1}{2}\begin{pmatrix} \cosh(2(r-r_{\mathrm{nl}})) & 0 & -\sinh(2(r-r_{\mathrm{nl}})) & 0 \\ 0 & \cosh(2(r-r_{\mathrm{nl}})) & 0 & \sinh(2(r-r_{\mathrm{nl}})) \\ -\sinh(2(r-r_{\mathrm{nl}})) & 0 & \cosh(2(r-r_{\mathrm{nl}})) & 0 \\ 0 & \sinh(2(r-r_{\mathrm{nl}})) & 0 & \cosh(2(r-r_{\mathrm{nl}})) \end{pmatrix}, \tag{125}$$

$$\boldsymbol{C} = \begin{pmatrix} 0 & \cosh(r_{\mathrm{nl}}) & 0 & -\sinh(r_{\mathrm{nl}}) \\ -\cosh(r_{\mathrm{nl}}) & 0 & -\sinh(r_{\mathrm{nl}}) & 0 \\ 0 & -\sinh(r_{\mathrm{nl}}) & 0 & \cosh(r_{\mathrm{nl}}) \\ -\sinh(r_{\mathrm{nl}}) & 0 & -\cosh(r_{\mathrm{nl}}) & 0 \end{pmatrix}. \tag{126}$$

The maximum sensitivity in the nonlocal MAI strategy is

$$\xi_{\mathrm{MAI,nl}}^{-2} = e^{2r}, \tag{127}$$

which is independent with the MAI-dependent squeezing parameter r_{nl}.

In local MAI strategies, local evolutions performed by one-mode squeezing operators $U_{\mathrm{loc}}^{(1)} = S_A = e^{r_{\mathrm{loc}}(a^2 - a^{\dagger 2})/2}$, $U_{\mathrm{loc}}^{(2)} = S_B = e^{r_{\mathrm{loc}}(b^2 - b^{\dagger 2})/2}$ are considered. The vector of MAI operators reads $\mathcal{A}_{\mathrm{MAI}} = \left(S_A^\dagger x_A S_A, S_A^\dagger p_A S_A, S_B^\dagger x_B S_B, S_B^\dagger p_B S_B\right)$, and the resulting covariance and commutator matrix are

$$\mathbf{\Gamma} = \frac{1}{2}\begin{pmatrix} e^{2r_{\mathrm{loc}}}\cosh(2r) & 0 & -e^{2r_{\mathrm{loc}}}\sinh(2r) & 0 \\ 0 & e^{-2r_{\mathrm{loc}}}\cosh(2r) & 0 & e^{-2r_{\mathrm{loc}}}\sinh(2r) \\ -e^{2r_{\mathrm{loc}}}\sinh(2r) & 0 & e^{2r_{\mathrm{loc}}}\cosh(2r) & 0 \\ 0 & e^{-2r_{\mathrm{loc}}}\sinh(2r) & 0 & e^{-2r_{\mathrm{loc}}}\cosh(2r) \end{pmatrix}, \tag{128}$$

$$\boldsymbol{C} = \begin{pmatrix} 0 & e^{-r_{\mathrm{loc}}} & 0 & 0 \\ -e^{r_{\mathrm{loc}}} & 0 & 0 & 0 \\ 0 & 0 & 0 & e^{-r_{\mathrm{loc}}} \\ 0 & 0 & -e^{r_{\mathrm{loc}}} & 0 \end{pmatrix}. \tag{129}$$

The maximum sensitivity after the measurement optimization is

$$\xi_{\mathrm{MAI,loc}}^{-2} = e^{2r}. \tag{130}$$

It is observed that the sensitivities in Eqs. (124)(127)(130) are the same, which solely depend on the initial squeezing parameter r.

To show the advantage from MAI technique in a realistic scenario, we consider the effect of detection noise. In this case, the measurement is written as $\tilde{x}^\alpha = x^\alpha + \Delta x^\alpha$ and $\tilde{p}^\alpha = p^\alpha + \Delta p^\alpha$, where ΔX is a random variable following the Gaussian distribution with mean value $\langle \Delta X \rangle = 0$ and variance $\langle (\Delta X)^2 \rangle = \sigma^2$. These detection noises will introduce additional errors to the detected variance, by adding an extra noise term to the covariance matrix via $\mathbf{\Gamma}' = \mathbf{\Gamma} + \mathbf{\Gamma}_\sigma$, with $\mathbf{\Gamma}_\sigma = \sigma^2 \mathbb{I}_4$. But it will not affect the mean values of detection statistics, leaving the commutator matrix $\boldsymbol{C}$ unchanged.

Influenced by detection noise, the sensitivity from the typical protocol becomes

$$\xi_{\mathrm{L}}^{-2} = \frac{1}{e^{-2r} + 2\sigma^2}. \tag{131}$$

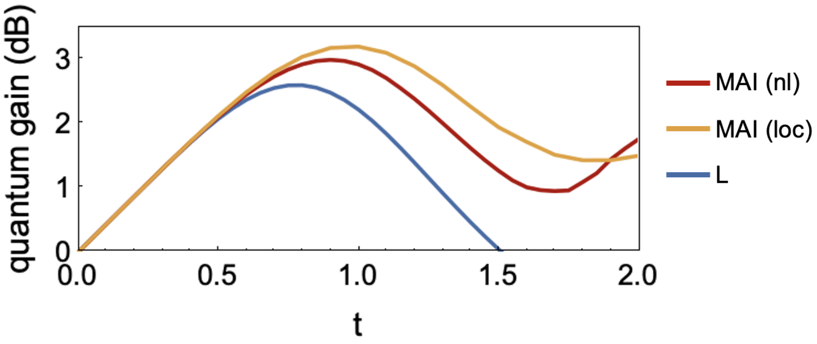

Supplementary Figure 4. Multiparameter metrological gain for two-mode squeezed-Kerr states $|\psi_{\text{Kerr}}\rangle$ under different readout strategies. We set $g = 0.5i$, $K = 2$ and plot sensitivities as a function of t, after optimizing the MAI evolution times τ.

In MAI protocols, the MAI operator under the detection noise is $U_\alpha^\dagger \tilde{X} U_\alpha = U_\alpha^\dagger X U_\alpha + \Delta X$. We obtain the sensitivity from the MAI protocols with nonlocal and local squeezing operators as

$$\xi_{\text{MAI,nl}}^{-2} = \frac{1}{e^{-2r} + 2e^{-2r_{\text{nl}}}\sigma^2}, \tag{132}$$

$$\xi_{\text{MAI,loc}}^{-2} = \frac{1}{e^{-2r} + 2e^{-2r_{\text{loc}}}\sigma^2}. \tag{133}$$

We demonstrate that the MAI protocols show improved noise robustness as long as $r_\alpha > 0$. The additional evolutions in MAI protocols effectively anti-squeeze the probes before the final measurements, rescaling the contribution of the detection noise in the measured quadratures. This noise analysis can be extended to general Gaussian probe states.

B. Multiparameter squeezing for two-mode squeezed Kerr states

Highly squeezing carries large excitation energy, and once this exceeds a platform's linear regime, inevitable nonlinearities drive the dynamics into a non-Gaussian regime. For a two-mode systems, the interplay between the squeezing and nonlinear interaction can be investigated with a squeezed-Kerr Hamiltonian

$$\tilde{H}_{\text{Kerr}} = \hbar\left[g\left(ab - a^\dagger b^\dagger\right) - K a^{\dagger 2} a^2\right], \tag{134}$$

where g is the squeezing parameter, and K is the Kerr nonlinearity. A pure non-Gaussian state, two-mode squeezed Kerr state, is generated by applying this Hamiltonian to a vacuum state, $|\psi_{\text{Kerr}}\rangle = e^{-i\tilde{H}_{\text{Kerr}} t/\hbar}|0\rangle$. Then, two displacement amplitudes g_m are locally encoded to the probes by phase generators G_m, yielding $e^{-i(G_1 d_1 + G_2 d_2)}|\psi_{\text{Kerr}}\rangle$.

For readout strategy, we still consider a complete set of quadrature observables $\mathcal{L} = (x, p)$. In nonlocal MAI protocols, we naturally use the same interaction as the one in state preparation,

$$U_{\text{nl}} = \exp\left[-i\tau\left(g(ab - a^\dagger b^\dagger) - K a^{\dagger 2} a^2\right)\right]. \tag{135}$$

In local MAI protocols, self-Kerr interactions are applied on two nodes,

$$U_{\text{loc}}^A = \exp\left[i\tau K a^{\dagger 2} a^2\right], \tag{136}$$

$$U_{\text{loc}}^B = \exp\left[i\tau K b^{\dagger 2} b^2\right]. \tag{137}$$

Here, τ is the MAI evolution time.

In Supplementary Figure 4, we present metrological gain detected under three detection strategies. As t increases, the sensitivity detected by typical linear measurement decays rapidly. While both MAI protocols are able to detect non-Gaussian states in a larger region.

[1] M. Kitagawa and M. Ueda, Phys. Rev. A **47**, 5138 (1993).

[2] Y. Baamara, A. Sinatra, and M. Gessner, Phys. Rev. Lett. **127**, 160501 (2021).
[3] M. Walschaers, S. Sarkar, V. Parigi, and N. Treps, Phys. Rev. Lett. **121**, 220501 (2018).
[4] Y.-S. Ra, A. Dufour, M. Walschaers, C. Jacquard, T. Michel, C. Fabre, and N. Treps, Nat. Phys. **16**, 144 (2020).
[5] J. Davis, M. Kumari, R. B. Mann, and S. Ghose, Phys. Rev. Res. **3**, 033134 (2021).
[6] A. Kenfack and K. Życzkowski, J. Opt. B: Quantum Semiclass. Opt. **6**, 396 (2004).
